# Geminin Is Required for the Maintenance of Pluripotency

**DOI:** 10.1371/journal.pone.0073826

**Published:** 2013-09-19

**Authors:** Golnaz A. Tabrizi, Kerstin Böse, Yvonne Reimann, Michael Kessel

**Affiliations:** Max Planck Institute for Biophysical Chemistry, Göttingen, Germany; Baylor College of Medicine, United States of America

## Abstract

Pluripotency requires the expression of the three core transcriptions factors Oct4, Sox2 and Nanog, as well as further, complementary proteins. The geminin protein is part of this network, and was shown to play a role in the regulation of DNA replication, the control of the cell cycle, and the acquisition of neural fate. It is highly expressed in the early embryo, in particular the epiblast and the early neural ectoderm, and also in pluripotent embryonic stem cells. The genetic inactivation of geminin resulted in lethality after the first few cell divisions, and thus prohibited the outgrowth of pluripotent cells. We established embryonic stem cells allowing the deletion of the geminin gene by induction of of Cre-recombinase with tamoxifen. Here, we show that geminin deficiency quickly leads to a loss of pluripotency, and to differentiation into the mesendodermal direction with high Oct4/low Sox2 levels. Simultaneous loss of geminin and induction of the neural lineage resulted in immediate apoptosis. These results suggested that in early development geminin functions via the co-expressed Sox2 gene. We found that the stem cell enhancer SRR2 of *Sox2* is occupied by the activating esBAF complex in the presence of geminin, but becomes epigenetically repressed in its absence by the Polycomb repressive complex PRC2. The importance of geminin for Sox2 expression also explains the absolute requirement for geminin during the induction of pluripotency by OSKM viruses. In summary, geminin is required for Sox2 expression, and thus for the maintenance of totipotency, pluripotency and the early neural lineage.

## Introduction

The developmental potential of cells in early mouse embryogenesis becomes systematically restricted, going down from toti-, to pluri-, multi- and unipotency. The totipotent oocyte and morula lose the capacity for extraembryonic tissue formation, so that the cells of the inner cell mass (ICM) and the early epiblast are only pluripotent, i.e. capable of forming only embryonic tissues. The pluripotent mouse embryonic stem cells (ESCs) resemble the ICM of the early blastocyst in terms of expression of core pluripotency gene network, such as Oct4, Sox2 and Nanog. These key transcription factors maintain the expression of each other and other pluripotency genes, through a crosstalk with chromatin remodeling complexes, governing the pluripotent state of the ES cells [1]. The interplay between Oct4 and Sox2 is a key regulatory mechanism in the establishment of the pluripotency and fate allocation [2]. Their expression diverges when either the neural/non-neural ectodermal, or the mesendodermal lineage is established at the onset of gastrulation. While Oct4 expression in the absence of Sox2 is crucial for acquisition of the mesendodermal fate, early neurogenic fate requires the presence of Sox2 in the absence of Oct4 [3].

A protein exerting multiple functions via protein binding of its coiled-coil domain is geminin. Its overexpression converts ectodermal progenitors into early neural cells, and thus can expand the neural plate during embryogenesis [4]. Most probably its involvement in fate decisions during development occurs through an influence on the epigenetic signature of neurogenic genes, activating early genes and suppressing later, neuronal differentiation genes [5]–[7]. Interactions of geminin with components of the Polycomb complexes and with chromatin modifiers such as the BAF complex were previously documented [8]–[10]. Geminin also functions in the regulation of replication [11]. It prevents the formation of pre-replication complexes by binding the replication licensing factor Cdt1, inhibiting its binding to origins, and preventing the firing of origins during the S and G2 phases of the cell cycle. In order to maintain genomic integrity, geminin and Cdt1 levels are highly balanced, and aberrations predispose a cell to malignant transformation [12]. Re-replication events can be prevented by a number of different mechanisms, when compensatory mechanisms are activated to block precocious replication, so that a stable genome can be maintained also in the absence of geminin [13].

Genetic inactivation of geminin in mice led to an arrest of development after the 8-cell stage [14], [15]. Remaining cells underwent endoreduplication, acquired a trophoblastic fate, and failed to grow out in culture to become ESCs. Conditional inactivation of geminin in various embryonic or adult cell types led to relatively minor phenotypes. Thus, thymocytes developed and differentiated normally in vivo in the absence of geminin, but required it after challenge in culture [16]. Geminin deficiency during hematopoiesis led to anemia [17]. Spermatogonial cells required geminin for mitotic division, but not for differentiation or meiosis of spermatocytes [18]. Minor defects were found in the development of the cerebral cortex in mice lacking geminin ([19], Reimann, Zeddies et al., unpublished data). On the other hand, neural stem cells, as well as early mesodermal cells divided and developed normally without geminin ([20], Reimann, Zeddies et al., unpublished data).

Geminin is ubiquitously expressed in embryonic tissues, and particularly high levels were found in ESCs and embryonal carcinoma (EC) cells [21]–[23]. These cells offer the possibility to study the establishment, acquisition and maintenance of early developmental fates in culture. Knock-down of geminin resulted in a loss of stem cell identity [22], and prohibited the neural fate acquisition by facilitating a hyper-acetylated state of the chromatin [6]. Additionally it resulted in the differentiation into a trophoblast-like cell type in EC cells but extraembryonic differentiation was not as clear in ESCs as in EC cells [22]. It was suggested that geminin functions in the regulation of Sox2 in the avian neural plate [9].

In this study we describe the conditional deletion of the geminin gene in ESCs grown either in stem cell or in neurogenic medium. Geminin turned out to be absolutely essential for the maintenance of pluripotent ESCs, or for the early neurogenic lineage. Fibroblasts could not be reprogrammed into pluripotency in the absence of Geminin, which was required in the late phase of pluripotency induction, similar to the endogenous Sox2 gene. We show that in geminin-deficient cells the downstream, stem cell specific enhancer of Sox2 becomes repressed by a prominent repressive histone mark. Our findings explain the requirement of pluripotent and early neural cells for geminin.

## Results

### Geminin protein is highly expressed in embryonic stem cells and neuroectodermal progenitors

**Figure 1:**
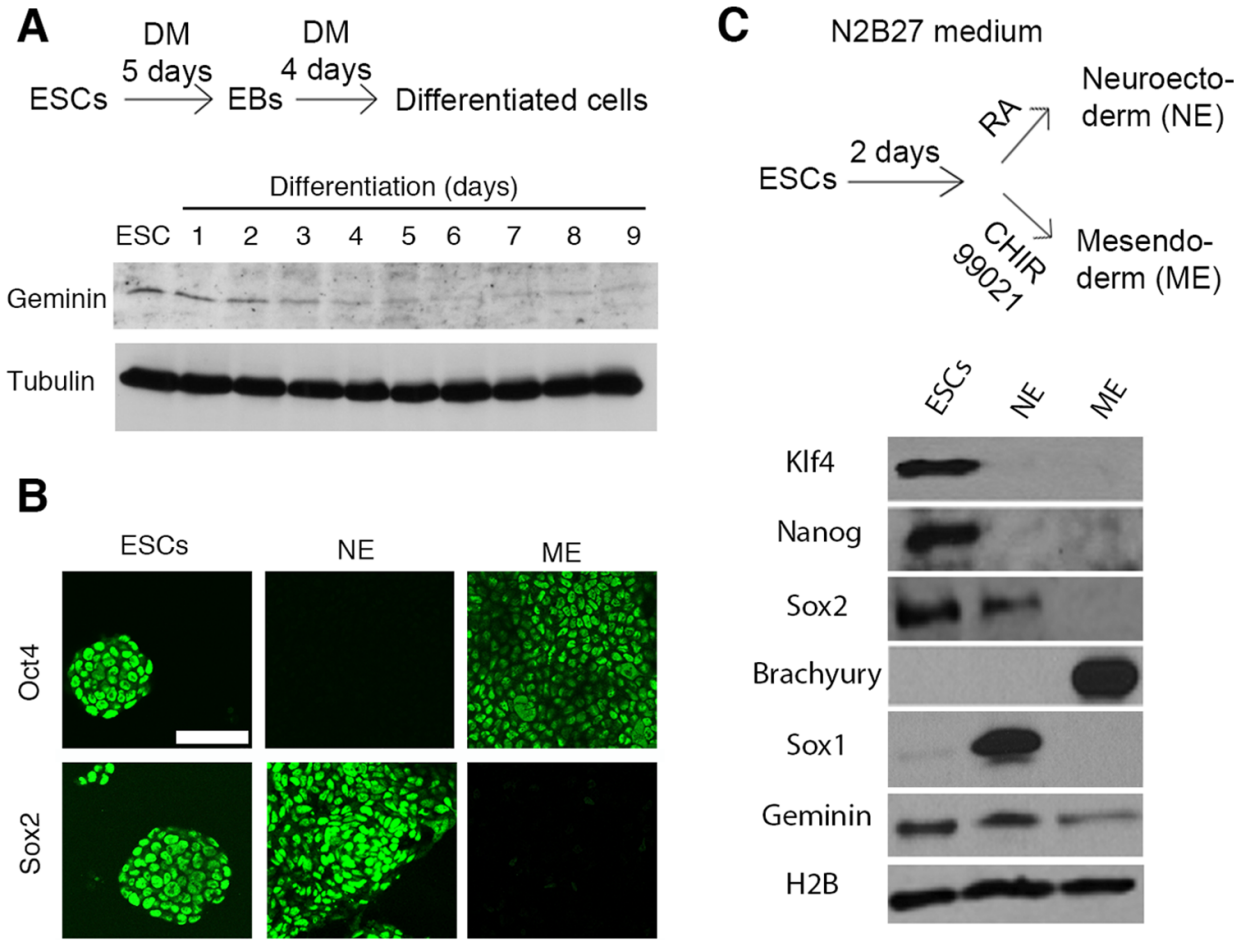
Down regulation of geminin, Oct4 and Sox2 in differentiation and fate acquisition. A) MPI-II ESCs were differentiated for 5 days as embryoid bodies followed by re-plating in adhesive culture plates for 4 more days to form the differentiated monolayer cultures. Whole cell lysates were harvested and analyzed by western blot. The amount of the loaded protein was controlled by α-tubulin amounts. **B**) MPI-II ESCs were differentiated on gelatin-coated plates in the absence of serum for 48 hours and then exposed to retinoic acid (RA) or GSK3β inhibitor, CHIR99021, in order to differentiate the ES cells to neuroectoderm (NE) and mesendoderm (ME), respectively. Undifferentiated ESCs, NE and ME were analyzed with immunofluorescence staining of pluripotency markers (Sox2 and Oct4), **C**) Western blot analysis of pluripotency markers (Sox2, Klf4 and Nanog), lineage specific markers (Sox1 and Brachyury) and Geminin. Histone 2B (H2B) levels are shown for control.

We examined the geminin levels in wild type mouse ESCs and their differentiating progenies. Whole cell lysate protein analysis showed that undifferentiated ESCs expressed geminin strongly, and the levels decreased during differentiation under standard conditions involving the formation of embryoid bodies (Fig. 1A). Pluripotency was confirmed for ESCs by the combined expression of Oct4, Sox2, Klf4, Nanog and Geminin (Fig. 1B,C). They were specifically differentiated towards the neuroectodermal or the mesendodermal lineage, which were identified by Sox1 or Brachyury, respectively (Fig. 1C). The simultaneous expression of Sox2 and Oct4 typical for ESCs diverged upon differentiation. Sox2 was only maintained in the neuroectodermal lineage, while it completely disappeared from mesendodermal cells (Fig. 1B,C). Oct4, on the other hand, was preferentially downregulated in neuroectoderm, and higher levels remained in mesendoderm. Geminin protein levels were higher in neuroectoderm than in mesendoderm progenitors (Fig. 1C).

### ESCs cannot self-renew after geminin deletion

**Figure 2:**
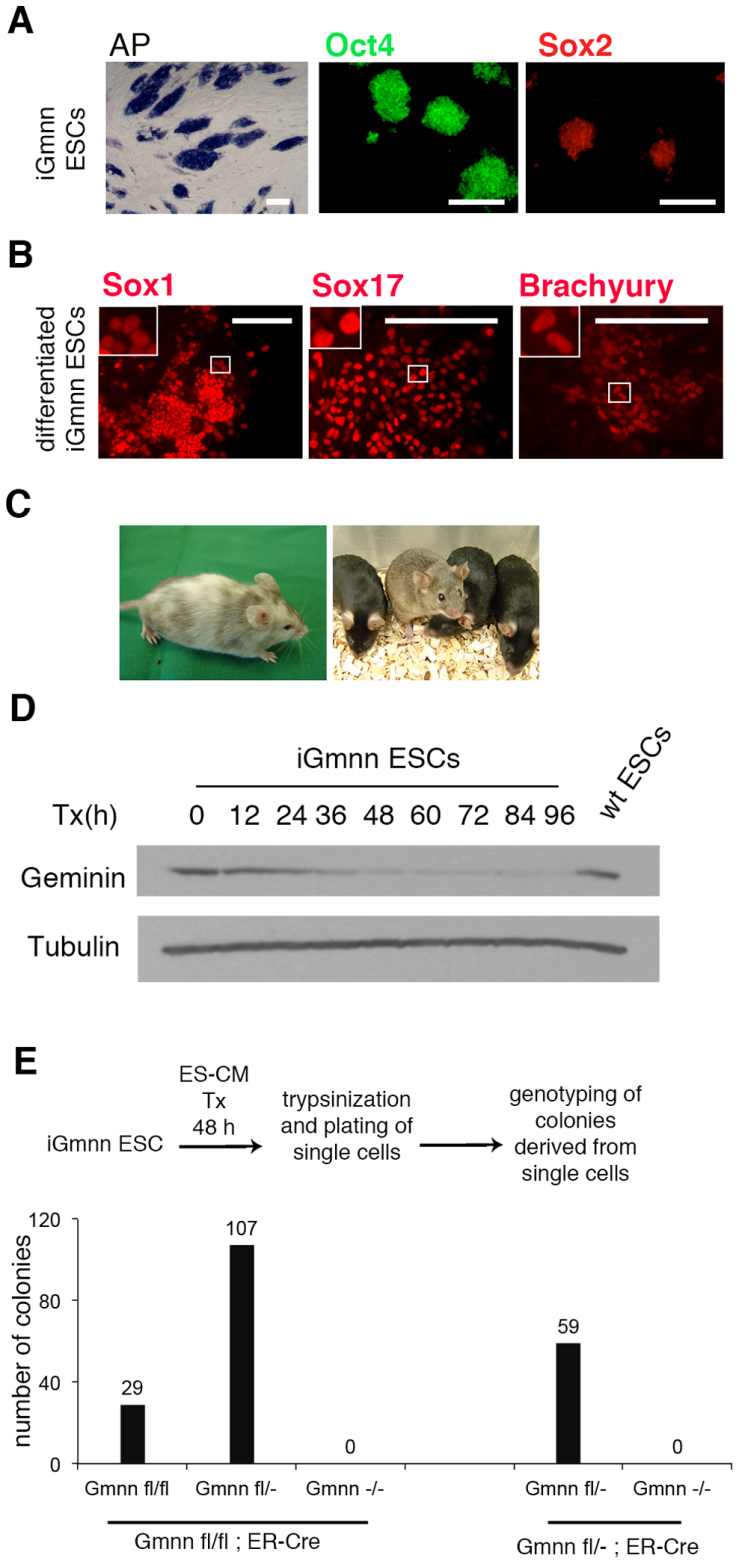
Geminin is essential for the self-renewal of ESCs. A) iGmnn ESCs were stained for alkaline phosphatase activity and were immuno-stained for the pluripotency markers, Oct4 and Sox2. **B**) iGmnn ESCs were differentiated for 9 days as floating EBs in petri dishes, followed by re-plating on tissue culture plates. Differentiated iGmnn ESCs were immuno-stained for lineage specific differentiation markers (Sox1: ectoderm marker, Brachyury: mesoderm marker and Sox17: endoderm marker). Boxed areas show magnifications of the nuclear staining for the transcription factors. **C**) Chimeras were driven from iGmnn ESCs and the ESCs were germ line transmissible. **D**) iGmnn ESCs were cultured for 96 hours in ES-CM, and were treated for indicated time periods with tamoxifen. The whole cell lysates were harvested and analyzed by western blotting. **E**) iGmnn ESCs were treated with tamoxifen and were trypsinized into single cells. The cells were grown on feeder-coated plates in order to give rise to single-cell derived clones. These clones were expanded and their genomic DNA was extracted. Genomic DNA samples from the grown ESC clones were analyzed by genotyping PCR. Three partially recombined Gmnn^fl/−^ clones were re-exposed to tamoxifen and trypsinized into single cells. The cells were grown to give rise to single-cell derived clones. These clones were expanded and genotyped by PCR.

To study the role of geminin in ESCs we generated a conditional knockout mouse in which the *Gmnn* exons 2 and 3 were flanked by loxP sites, and thus were excisable upon treatment with Cre recombinase, generating a nonfunctional knockout allele (Fig. S1A-B). The conditional knockout mice were bred to ER-Cre mice, expressing ubiquitously tamoxifen inducible Cre recombinase [24]. Blastocysts were cultured, established ESC lines were genotyped, and one line with the desired *Gmnn* ^fl/fl^ *ER-Cre* genotype, designated iGmnn, was selected for further characterization. Colony morphology, alkaline phosphatase activity, and expression of the pluripotency markers Oct4 and Sox2 confirmed the stem cell characteristics of the iGmnn cells (Fig. 2A). Their differentiation potential was demonstrated by the ability to give rise to all three germ layers, i.e. neuroecto-, endo- and mesoderm, as identified by Sox1, Sox17, or Brachyury expression, respectively (Fig. 2B). The iGmnn ESCs contributed extensively to tissues of chimeric mice, including the germline (Fig. 2C). Together, these characteristics demonstrated that the iGmnn ESCs represent a fully pluripotent cell line. Next we tested the efficiency of tamoxifen-induced, Cre mediated homologous recombination in deletion of the geminin allele(s). Genotyping (Fig. S1C) and western blot analysis (Fig. 2D,S1D) of cells treated for different periods of time revealed an efficient recombination and removal of Geminin, after 48 hours of exposure to 1 μM tamoxifen. Tamoxifen at this concentration was not toxic for wild-type or Gmnn^fl/+^ ESCs.

iGmnn ESCs were treated with tamoxifen while being cultured in conventional embryonic stem cell medium (ES-CM). After 48 hours, cells were trypsinized, and plated on feeder layers. After a few days, colonies were sub-cloned and expanded. Genotyping of these colonies revealed that 29 *Gmnn* ^fl/fl^ unrecombined clones and 109 *Gmnn* ^fl/−^ heterozygotes, but not a single *Gmnn* ^−/−^ knockout colony had been established. To exclude the possibility of incomplete recombination we treated 3 of heterozygous *Gmnn* ^fl/−^ colonies with tamoxifen once more and sub-cloned the resulting cells again. The inability of geminin deficient cells to self-renew and form a colony, was confirmed by the genotype analysis of the obtained 59 *Gmnn* ^fl/−^ cell lines (Fig. 2E). Taken together this analysis shows that geminin is necessary for the maintenance and self-renewal of pluripotent ESCs in culture.

### Geminin is required for the acquisition of a neural fate

**Figure 3:**
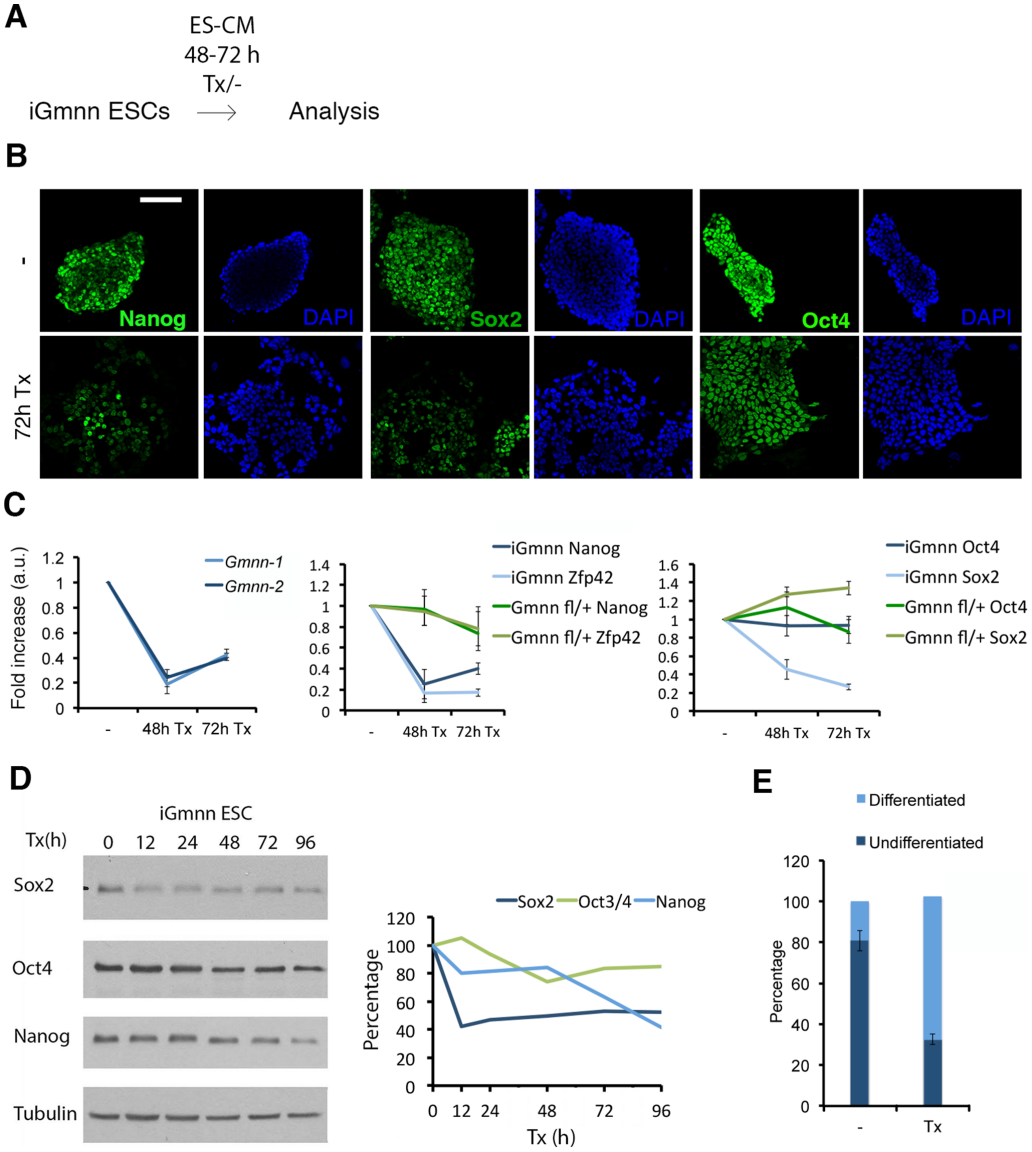
Geminin is essential for the pluripotent state of ESCs. A) iGmnn ESCs were treated with tamoxifen for 48/72 hours. **B**) iGmnn ESCs were treated with tamoxifen for 72 hours and immunostained for pluripotency markers. The white bar represents 100 μm. **C**) iGmnn ESCs and Gmnn^fl/+^ ESCs were treated with tamoxifen for 48 and 72 hours, harvested for RNA extraction and mRNA was analyzed by quantitative RT-PCR. The mean Ct value were calculated and normalized to the expression of Gapdh and Hprt. Relative enrichment of transcripts was calculated in comparison to untreated cells. Error bars represent ± standard error of the mean (SEM) of technical triplicates. **D**) iGmnn ESCs were cultured for 96 hours in ES-CM, and were treated for different time periods with tamoxifen. The whole cell lysates were harvested and analyzed by western blotting, and quantified with ImageJ. **E**) iGmnn ESCs were stained for alkaline phosphatase activity. The colonies were quantified according to their AP staining and morphology.

**Figure 4:**
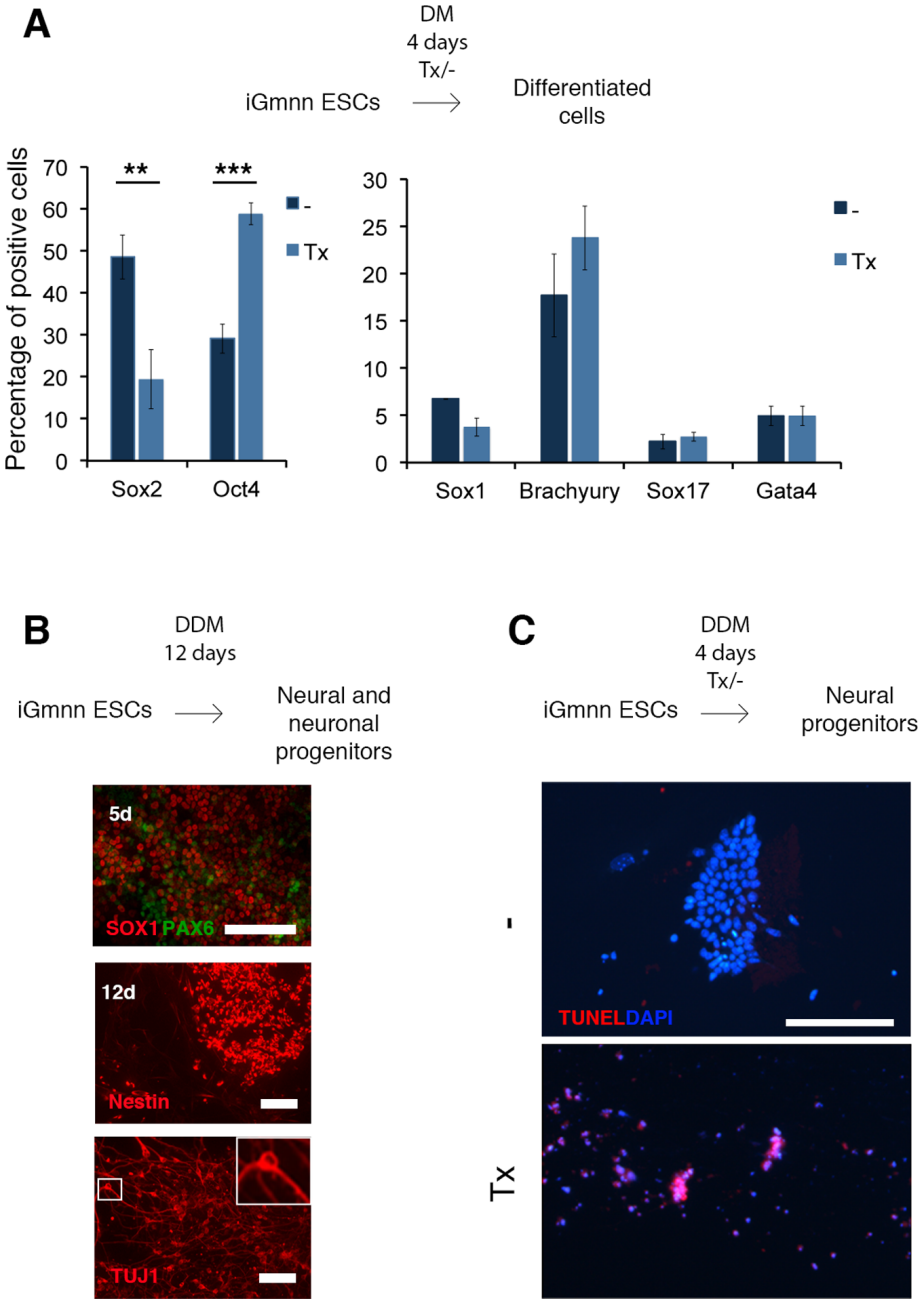
Geminin is necessary for the differentiation of ESCs to the neural lineage . A) iGmnn ESCs were differentiated in the differentiation medium (DM) and treated with tamoxifen for 4 days. The differentiated ESCs were immunostained for Sox2 and Oct4 and for the differentiation markers and quantified (Sox1: neural lineage, Brachyury: mesoderm, Sox17: Endoderm, Gata4: primitive endoderm). **B**) iGmnn ESCs were differentiated in the absence of tamoxifen to the neural lineage for up to 12 days, and stained for Sox1, Pax6, Nestin and Tuj1. A single Tuj1 expressing neuron is magnified in the box. **C**) iGmnn ESCs were differentiated to the neural lineage in the presence (lower panel) or absence (upper panel) of tamoxifen for 4 days and stained for TUNEL activity. Genomic DNA was stained with DAPI.

iGmnn ESCs were grown under stem cell promoting conditions, treated with tamoxifen for 48 or 72 hours in ES-CM on feeder layers, and analyzed further (Fig. 3A). The number of colonies after tamoxifen treatment was reduced slightly, and the proliferation rate of the treated cultures was lower than in untreated cells. There was no difference in the number of mitotic and apoptotic cells (Fig. S2A). An investigation of the cell cycle phases in treated versus untreated cells revealed a slight increase in the cell numbers in the G1 phase cells at the expense of cells in the G2/M phase (Fig. S2B). Such a shift is characteristic for an ESC population that started to differentiate, suggesting that the tamoxifen exposed iGmnn cells began to diverge from the stem cell status. Their further characterization indicated a down-regulation of the pluripotency markers Nanog and Sox2, but not Oct4 (Fig. 3B). Similar results were observed by qPCR (Fig. 3C) and western blot analysis (Fig. 3D), in which mRNA and protein levels of the pluripotency genes Nanog, Zfp42 (Rex1) and Sox2 were down regulated in the tamoxifen treated cells, while Oct4 levels remained at the level detected in the untreated cultures. Control experiments with heterozygous ESCs (Gmnn^fl/+^) indicated that the effects were specific for the absence of geminin, and were not an unspecific consequence of tamoxifen treatment (Fig. 3C). After 72 hours of tamoxifen treatment the flattened and dispersed colony morphology indicated ongoing differentiation. Based on alkaline phosphatase activity and morphology the colonies were categorized either as differentiated and non-pluripotent, or as undifferentiated and ES-like (Fig. 3E). Quantification of colonies depicted a significant (p-value <0.0001) shift toward differentiated cell morphology after tamoxifen treatment. In order to identify the lineage of the differentiating cells we applied markers for trophectoderm (Cdx2, Troma-I), the neuroectodermal (Pax6, Nestin, Sox1), and the mesendodermal lineage (Gata4, Bachyury, Sox17), none of which was detectable (Fig. S3A-C). In a second approach, we removed geminin from cells grown under differentiation promoting conditions, i.e. high serum (20%) in the absence of LIF. After 96 hours of tamoxifen exposure, we observed a significant increase in the percentage of Oct4 positive cells (p-value 0,0004), and a corresponding decrease in the percentage of Sox2 expressing cells (p-value 0,0084, Fig. 4A, left panel). In both the tamoxifen treated and the untreated cell populations the expression of Sox1, Brachyury, Sox17, and Gata4 was detected in a fraction of cells. However, the percentages of positive cells did not differ significantly after deletion of geminin (p-values 0.0905, 0.581, 0.823, 0.3023, respectively; Fig. 4A right panel). No Cdx2 or P-cadherin positive cell was detected in the cultures, so that no indication for trophectoderm differentiation was obtained (data not shown). As a third type of growth condition we applied a medium [25], which would normally drive ESCs into a neural fate (Fig. 4B). Here, tamoxifen treated iGmnn ESCs underwent apoptosis, and already after 4 days basically no cell had survived the treatment (Fig. 4C). Taken together, it became clear that ESCs lost their stem cell characteristics quickly after the deletion of geminin. In differentiation promoting conditions the absence of geminin led to a downregulation of Sox2, and the simultaneous maintenance of Oct4 levels, indicating a differentiation into the mesendodermal, rather than the neuroectodermal direction. For differentiation of ESCs into the early neural lineage the maintenance of a high Geminin level was absolutely essential, possibly as a prerequisite for the expression of Sox2.

### Geminin regulates *Sox2* expression through chromatin modifications

**Figure 5:**
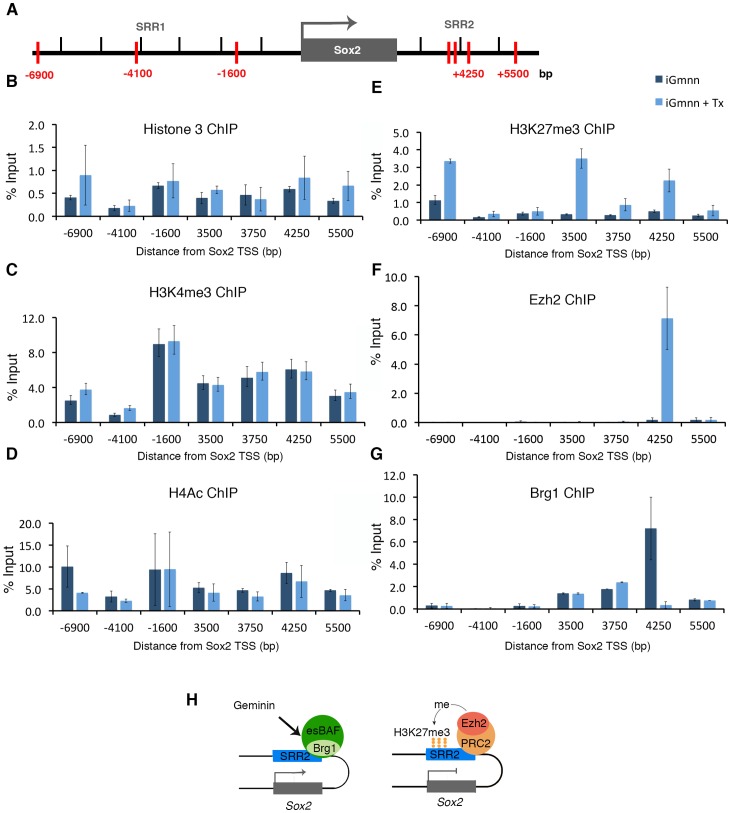
Geminin regulates the epigenetic signature of Sox2 enhancer region. A-G) ChIP-qPCR assays epigenetic marks binding at genomic locus of sox2 gene. A) Sox2 genomic locus, analyzed fragments of the DNA have been marked with red, SRR1: stem cell regulatory tegion 1, SRR2: stem cell regulatory region 2. Histone 3 ChIP (B), histone 3 lysine 4 tri-methylation (H3K4me3) ChIP (C), Histone 4 hyper-acetylation (H4Ac) ChIP (D), Histone 3 lysine 27 tri-methylation (H3K27me3) ChIP (E), Ezh2 ChIP(F) and Brg1 ChIP (G) in tamoxifen treated cells iGmnn cells and untreated iGmnn ESCs. Each sample is normalized to input, and error bars represent ± standard error of the mean (SEM) of biological (B, D-G) or technical (C) triplicates. The X-axis represents positions relative to the transcriptional start site. **H**) The chromatin at the SRR2 enhancer downstream of Sox2 gene. The active enhancer is associated with the esBAF complex and requires the function of Geminin. Note that Geminin is not a part of the esBAF complex, and does not bind to Brg1 in ESCs. In the absence of Geminin SRR2 becomes inactive, and associates with the PRC2 complex, including its component Ezh2. Ezh2 catalyzes the tri-methylation of histone 3 on residue K27, and thus establishes a repressive epigenetic signature.

Since the described experiments point to a connection between geminin and the transcription factor Sox2, we performed chromatin immuno-precipitations (ChIPs) on the Sox2 and the Oct4 loci of tamoxifen-exposed or unexposed iGmnn ESCs. In particular, we checked the occupancy of the previously described stem cell regulatory regions of the Sox2 gene, the SRR1 and SRR2 enhancers [26] and the distal enhancer element, DE [27] of the Oct 4 gene, as well as intermittent regions (Fig. 5A,S4). The data indicate no significant change of total histone 3 in all analyzed regions after tamoxifen treatment (Fig. 5B,S4). The activating histone modifications H3K4me3 or H4Ac did not differ in geminin containing or deficient cells (Fig. 5C,D,S4). In contrast, the repressive histone mark H3K27me3 was specifically increased on the SRR2 enhancer after deletion of Geminin, in parallel to the responsible methyltransferase Ezh2, the catalytic component of the Polycomb repressive complex 2 (PRC2; Fig. 5E,F). The PRC2 and esBAF (stem cell specific mSWI/SNF) complexes compete for regulatory regions of pluripotency genes, including the enhancer regions of Sox2 and Oct4 [28]. We did not find an association of Sox2 or Oct4 enhancer sequences with geminin in ESCs. Neither did we detect an interaction between geminin and Brg1 by Co-IP (data not shown). ChIP analysis of Brg1, the core component of esBAF complex, indicated that it dissociates from the SRR2 enhancer in Geminin deficient cells, in exchange for the presence of Ezh2 (Fig. 5F,G). ChIP experiments done with anti-geminin antibody were not able to detect a significant binding of geminin to this region. Taken together, the ChIP analysis points to the SRR2 enhancer of the Sox2 gene as a regulatory site affected by the presence or absence of geminin (Fig. 5H).

### Geminin is necessary for the reprogramming of fibroblasts to pluripotency

**Figure 6:**
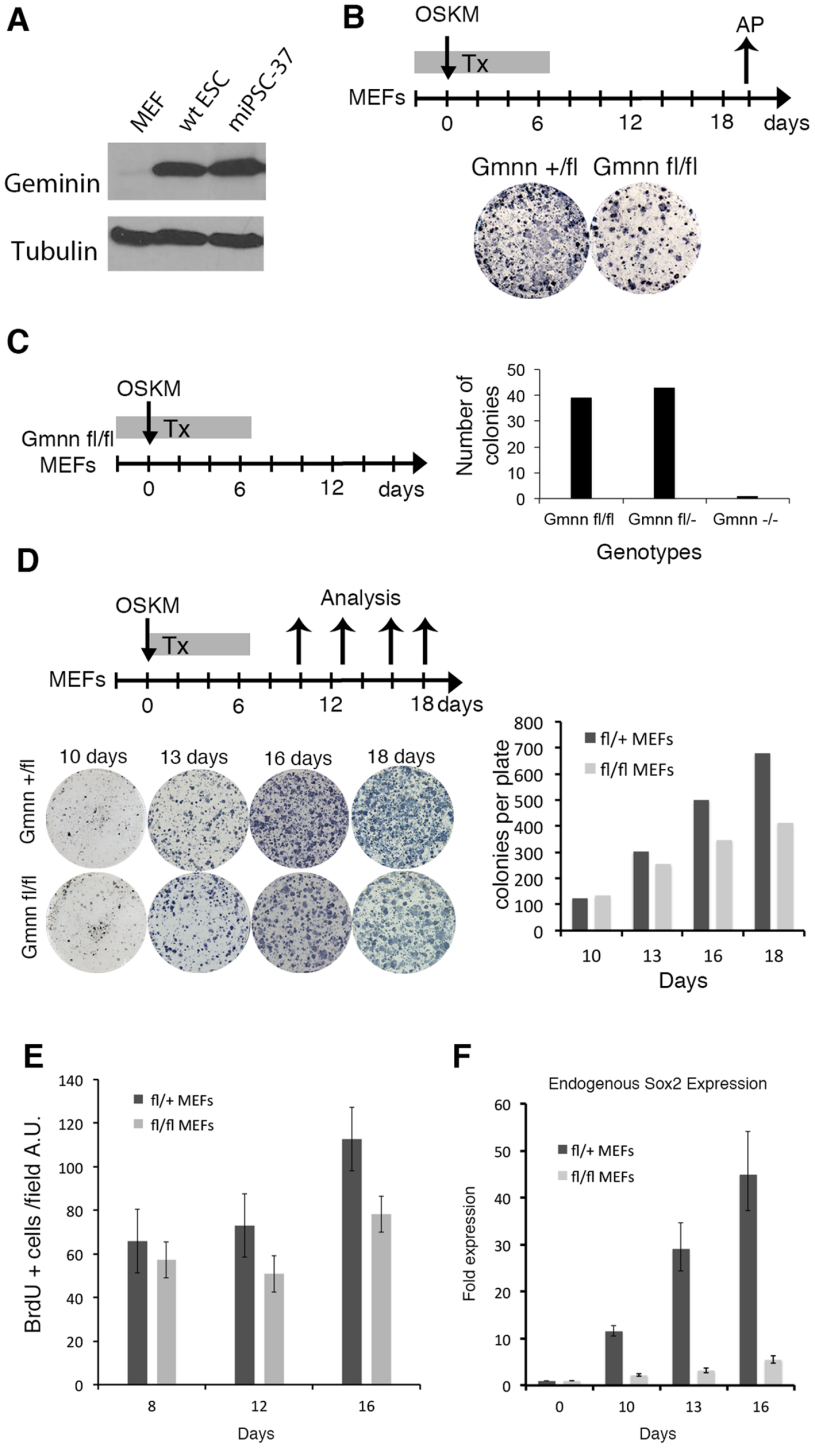
Geminin is necessary for the maintenance of reprogramming. A) fl/+ and fl/fl MEFs were reprogrammed with OSKM (Oct4, Sox2, Klf4 and C-Myc) viral particles in the presence of tamoxifen. Transduced plates were stained for alkaline phosphatase after 20 days. **B**) Reprogrammed fl/fl MEFs were sub-cloned and genotyped. Only one knockout line observed, showed a differentiated morphology and failed to grow further. **C**) Western blot analysis of MEFs, and the pluripotent cell lines MPI-II ESCs and iPSC-37. **D**) fl/+ and fl/fl MEFs were reprogrammed with OSKM viral particles in the presence of tamoxifen. Reprogrammed plates were stained for alkaline phosphatase at different time points. **E**) fl/fl and fl/+ MEFs were reprogrammed with OSKM viral particles in the presence of tamoxifen. At different time points, the transduced plates were treated with BrdU and stained for it. The graph represents the number of BrdU incorporated cell per counted field relative to the control cells. **F**) fl/fl and fl/+ MEFs were reprogrammed with OSKM viral particles in the presence of tamoxifen. At different time points, the amount of endogenous Sox2 mRNA has been quantified. The graph represents the fold increase of endogenous Sox2 relative to day0 MEFs.

*Gmnn^fl/fl^ ER-Cre* mouse embryonic fibroblasts (fl/fl MEFs) allow for the induction of Geminin deletion by tamoxifen treatment. After 48 hours the floxed Gmnn allele had recombined as confirmed by genotyping, and only small amounts of protein were detectable by western blot analysis (Fig. S5A). No significant morphological differences were detected between fl/fl and fl/+ MEFs after tamoxifen exposure. Flow cytometry of PI stained cells revealed no cell cycle aberrations (Fig. S5B). Comparable numbers of cells underwent mitosis, as marked by phosphorylated histone 3, and the same number of cells in the S phase labeled with a 4-hour bromo-deoxyuridine (BrdU) pulse (Fig. S5C). Cells were stained for well-known cell cycle markers and the abundances of positive cells were calculated as percentage of the total population. fl/fl MEFs contained same number of positive cells for cyclins (cyclin D1, A2 and B1) depicting that geminin knockout had not induced a cell cycle arrest in these cells (Fig. S5D). Ki67 expression, a marker for proliferating cells, was normal in the fl/fl MEFs indicating that the knockout cells are proliferating with a rate comparable to the fl/+ cells (Fig. S5E). In addition, TUNEL staining indicated no significant increase of apoptosis (Fig. S5F). Geminin levels were determined in wild type MEFs, ESCs and iPSCs (line iPSC-37) by western blotting of whole cell lysates (Fig. 6A). This analysis revealed significantly elevated geminin levels (more than 20 times) in the pluripotent cells.

Next we tested whether geminin is required for the induction of pluripotency. We used *Gmnn^fl/fl^ ER-Cre* MEFs or heterozygous controls, and followed established protocols applying retroviruses carrying the Oct4, Sox2, Klf4 and c-myc genes (OSKM) [29]. Tamoxifen was applied together with the addition of virus particles, and kept for a period of 7 days on the cells. AP staining of 20-day cultures revealed a significantly lower number of AP positive colonies in tamoxifen-exposed cultures to the control (Fig. 6B), which were then isolated and expanded. Genotyping revealed that no pluripotent clones had been isolated which lacked both Geminin alleles (Fig. 6C). Notably, the only derived *Gmnn* ^−/−^ cell clone, showed a differentiated morphology, delayed growth kinetics, and failed to passage. Analysis of alkaline phosphatase stained plates in the course of time, demonstrated that initial steps of reprogramming were intact in the absence of geminin but after nearly two weeks of reprogramming the number of AP positive colonies was reduced in the *Gmnn^fl/fl^* plates (Fig. 6D), indicating that the geminin deficient cells failed to maintain their pluripotent state. Quantification of BrdU positive cells in transduced fl/+ and fl/fl MEFs revealed a comparable number of positive cells at earlier stages, followed by a significant (p-value = 0.0232) decrease of proliferating cells at later stages. This observation is consistent with aberrant maintenance of the reprogramming in geminin deficient cells (Fig. 6E). It has previously been shown that a key process in this maintenance phase is the activation of endogenous pluripotency genes [30]. We followed the expression of endogenous Sox2 in cell populations undergoing reprogramming. In control (Gmnn^fl/fl^) cells the activation of the Sox2 gene in the maintenance phase was evident (Fig. 6F). The Geminin-deficient cell population generated by the exposure Gmnn^fl/fl^ fibroblasts to tamoxifen, on the other hand, did not increase their endogenous Sox2 levels (Fig. 6F).

By substituting separately each of the classical reprogramming factors by geminin it appeared, that none could be replaced by geminin for the efficient induction of pluripotency in fibroblasts (Fig. S6). It was previously reported that the small molecule RepSox can replace Sox2 in reprogramming by inducing Nanog [31]. However, the combination of OKM and RepSox was not sufficient to give rise to mature iPSCs from tamoxifen treated *Gmnn^fl/fl^* fibroblasts (data not shown). Together, these results demonstrate that pluripotency cannot be induced in fibroblasts in the absence of geminin.

## Discussion

### Replication and cell cycle regulation in the absence of geminin

Two main functions were identified in the two early studies reporting the discovery of the geminin gene: its role in regulation of replication licensing and its role in the acquisition of neural fate [4], [11]. In rapidly proliferating cells such as cleavage stage embryos, pre-implantation embryos and cancer cells, Cdt1 activity is the rate-limiting factor for the origin licensing [32]. Geminin binds and inactivates Cdt1 while protecting it from ubiquitination and degradation. Therefore in fast proliferating cells such as ES cells, geminin deficiency would result in a loss of Cdt1 [33]. In two knockdown studies [6], [33], no re-replication was observed while in contrast Yang and colleagues observed nuclei enlargement in ESCs [22]. This discrepancy could result from different residual levels of geminin after siRNA depletion. In our ESCs the inactivation of the geminin gene did not lead to re-replication, but resulted in a slightly longer cell cycle. Geminin down-regulation may have caused in a slower cell cycle, and this change led to the loss of the pluripotent identity. It is widely accepted that a fast, abbreviated cell cycle is necessary for the pluripotency of the ESCs [23], [34]–[36]. Noteworthy, it was shown that upon cell cycle perturbations or depletion of some cell cycle regulators the pluripotency markers are still up regulated debating the connection between the fast cell cycle and the pluripotent identity [37]. On the other hand an induction of differentiation and reduction in the pluripotency gene expression, can lengthen the cell cycle of the ESCs [23], [38], [39]. In summary, we interpret the observed increased length of the cell cycle after geminin knockout as a consequence of differentiation of ESCs.

### Epigenetics and fate acquisition in the absence of geminin

Non-genetic evidence by knockdown or overexpression has established the importance of geminin in the acquisition of fates early in embryonic development of frogs, and in the differentiation of murine ESCs and embryonal carcinoma cells. Here, a modulation of geminin levels affected the epigenetics and consequently also the transcriptional status. Partially, such effects were explained by the binding of geminin to key components of major chromatin modifying complexes, such as Brg1 from the BAF complexes, or the Scmh1 and Mph1 proteins from the Polycomb complexes [5], [8], [10]. Our own Co-IP analysis gave no indication for an association of geminin and Brg1 in ESCs. This observation is in line with the analysis of stringently purified complexes from ESCs, such as esBAF, neural BAF, PRC1 or PRC2, which did not contain geminin [40]–[43]. Thus, it appears that geminin could only function as a transient member of chromatin modifying complexes, via interaction with components before complex formation, or rather be involved in the complex assembly process, promoting or inhibiting the formation of a finally either activating or repressing complex.

Geminin protein was previously found in the cytoplasm, nucleoplasm, and in significant amounts also in the chromatin fraction of cells [44]–[46]. Its detection by ChIP analysis required modified methodology, such as a double PCR strategy, or extra crosslinking with disuccinimidyl glutarate [9], [47]. The only published demonstration of geminin by standard ChIP analysis refers to the transcriptional start sites of neural progenitor genes Pax6, Atoh1, Ebf2 and Sox1 in differentiating ESCs [6].

It was previously described that geminin mediates the expression of the pluripotency genes Oct4, Sox2 and Nanog by antagonizing Brg1 [22]. Our data rather point to an antagonistic effect of geminin on PRC2, which would keep the Sox2 enhancer active in the stem cells. Sox2 loci in ESCs are normally associated with esBAF/Brg1, but not PRC2 (Fig. 5H) [28]. It has been shown that these two chromatin remodeling complexes act both antagonistically and synergistically with the common goal of supporting pluripotency. esBAF is required to establish chromatin accessibility at pluripotency genes, and deletion of Brg1 leads to rapid Polycomb (PcG) binding and H3K27me3-mediated silencing of the target loci [48]. We detected drastic changes of the SRR2 chromatin in response to the genetic removal of geminin, which led to the exchange of esBAF with the PRC2 complex, as identified by its member, the histone 3 methyltransferase Ezh2. This would predict the methylation of histone 3 on residue K27, which was observed. In parallel to this repressive histone modification, the active, acetylated state of histone 4 was maintained after deletion of geminin. Thus, we conclude that geminin prevents the repression of the Sox2 enhancer by antagonizing PRC2 in ESCs, and is required for the maintenance of the transcription of the Sox2 gene under control of the SRR2 enhancer associated with the esBAF complex. Geminin affects the recruitment of the esBAF complex on the Sox2 loci and contributes to the active chromatin state of this gene. The switch from an active to a repressive chromatin modifying complex in dependence on geminin is depicted in the model in Fig. 5H.

### Pluripotency requires geminin for both replication and fate acquisition

Previous results suggested a requirement for geminin in totipotent cells of the morula, and thus made the genetic analysis of later stages impossible [14], [21]. Using a conditional knockout approach we could eliminate geminin completely from pluripotent and differentiating ESCs, and show that neither pluripotent stem cells nor early neural progenitor cells can be maintained in the absence of geminin. Our approach differed from knockdown approaches, which leave significant levels in some cells. Taken together it appears that the complete early axis of totipotent, pluripotent, and neurogenic cells requires geminin. These three cell types are characterized in addition to a high geminin expression, by high levels of the transcription factor Sox2. The mesendodermal lineage, on the other hand, down-regulates Sox2, maintains Oct4 expression, and thus segregates from the neural lineage [3]. Our data indicate that in pluripotent cells one effect of geminin is transduced via the modification of the Sox2 chromatin, in particular its stem cell enhancer SRR2. Very similar to geminin, Sox2 is required for the formation of the pluripotent ICM and the neuroectoderm, and is downregulated in the mesendodermal lineage [49]–[51]. Also, both genes have to be downregulated in order to allow neuronal differentiation later in development [5], [9], [52]. The expression of Sox2 is necessary for the maintenance and survival of pluripotent ESCs, and during reprogramming, its activation initiates consecutive steps that leads to the pluripotent state [50], [53], [54]. It is noteworthy that ESCs do not tolerate the overexpression of Sox2, and even small alterations trigger differentiation [55]. A perturbed balance between Sox2 and Oct4 would be expected to influence a large spectrum of downstream targets required for the establishment and/or maintenance of the pluripotency circuit. For these reasons it is problematic to rescue the deficiency for geminin by the exogenous expression of Sox2.

The absolute requirement of geminin for pluripotency suggests, that mutant fibroblasts cannot be reprogrammed to ESC-like cells. Our experiments revealed that the four factors OSKM initiate the induction of pluripotency in mutant fibroblasts with the same efficiency as in wild type cells. However, after around 10 days, the number of ESC-like colonies decreased, and the remaining colonies had not deleted their geminin alleles. This timing indicated that after an initial induction by retrovirus encoded factors, colonies could not maintain and stabilize their pluripotent features. Early in reprogramming, a stochastic gene expression allows an induction of pluripotency. However in later steps, a gene expression cascade initiated by Sox2 activation, leads to the maintenance of the pluripotent cells [54]. This activation fails in the absence of geminin, so that geminin deficient cells fail to re-express Sox2 and consequently to re-establish the pluripotency cascade. It appears likely that the same epigenetic mechanism found for ESCs also applies for iPSCs, namely the activity of the Sox2 enhancer depending on the presence of geminin, which decides on the association with the esBAf or the PRC2 complex.

## Materials and Methods

### Ethics statement

Animal experimentation and housing was performed in strict accordance with the law for animal welfare in Germany (TierSchG). The facility at the Max Planck Institute for Biophysical Chemistry is registered at the city of Göttingen under Az 32.22/Vo and Az 392001/7. The generation of chimeras is approved by the Niedersächsisches Landesamt Niedersachsen (Oldenburg) under Az 33.9-42502-04-11/0622. All surgery was performed under carprofen anesthesia, and all efforts were made to minimize suffering.

### Generation of conditional targeting construct, ES cell aggregation and chimera production [56]

A conditional targeting vector was generated by recombineering [57]. The genomic geminin locus was retrieved from the PAC clone RPCIP711F02244Q2 (imaGenes, mouse PAC-Bank (RPCI-21) Nr.711, Line 129S6/SvEvTac). The first loxP site was inserted 143 bps upstream of exon 2, the second loxP site, together with a frt-site flanked neomycin cassette, 182 bps downstream of exon 3. The HpaI-linearized targeting construct was electroporated into Sv129 ES cells, MPI-II [58], and clones were picked after double selection in G418 and ganciclovir. Proper integration of the targeting construct was checked by southern blotting after NdeI or StuI digestion with PCR amplified probes (primers for probe amplification: 5′ probe: 5′- GAGAAGCAAGCAAGCAAAC -3′ and 5′- GATTCAACGACGCCAGAACG-3′; 3′-probe: 5′- GCAGTAAGTTTCCCTATTGA GC-3′ and 5′ CACAGGTGAGTAGATCTGGTG-3′). Correctly targeted clone (Gmnn^fl/+^ ESCs) was aggregated with morula stage embryos from CD1 mice and re-implanted into the uteri of CD1 foster mothers. The resulting chimeric mice were further mated to CD1 mice for germline transmission.

### Cell culture

Gmnn^fl/fl^ ER-Cre and Gmnn^fl/+^ ER-Cre primary mouse embryonic fibroblasts (MEFs) were derived at E13.5 from appropriate crosses. MEFs were cultured in Dulbecco's Modified Eagle's Medium (DMEM, Gibco) supplemented with 10% fetal bovine serum, and passaged at sub-confluence. “iGmnn” ESCs were derived from 3.5 dpc blastocysts, 4–6 were cultured in embryonic stem cells conventional medium (ES-CM: Knockout™ DMEM (Gibco) supplemented with 20% fetal calf serum (FCS, PAN-biotech), 1 mM β-mercaptoethanol (Sigma-Aldrich), 2 mM L-Glutamine (Gibco), 1% non essential amino acids (Gibco), 1 mM sodium pyruvate (Gibco) and 1000 u/ml leukemia inhibitory factor (LIF, Invitrogen)) on feeder cell-coated 35-mm culture plates. The medium was changed every 2 days, and 5–6 days after plating the blastocysts had outgrown into ESCs. Outgrowths were cut, trypsinized and expanded into ES lines. ESCs (MPI-II, Gmnn^fl/+^ and iGmnn) were routinely maintained on feeder-coated 35-mm plates and fed daily with ES-CM. ESCs were passaged every 2–3 days depending on the level of confluency. Embryoid body (EB), monolayer, or lineage specific differentiations were induced as explained in text S1 [59]. In order to induce the recombination of Geminin alleles, 4-hydroxyl tamoxifen, “Tx”, (Sigma-Aldrich) was added to the fibroblast growth medium to a final concentration of 100–500 nM, and to the ES-CM medium to a final concentration of 1 μM.

### Antibodies for immunostaining

Primary antibodies were Brachyury, Sox1, Sox17 (diluted 1:100, R&D), Cyclin A2, Cyclin B1, Cyclin D, Gata4 (1:100), Geminin-FL209 (1:50), Sox2 (1:100), SSEA1 (1:300, Santa Cruz Biotechnologies), Ki67 (1:200, Abcam), Nanog (1:100, Cosmobio), Oct3/4 (1:200, BD Bioscience), Phospho-histone 3 (1:200, Cell signaling), Troma-I (1:100, DSHB), Sox2 (1:100, Millipore), Pax6 (1:100, Covance) and Cdx2 (1:100, Biogenex). Appropriate secondary antibodies labelled with Alexa fluorophores (Invitrogen) were diluted 1:1000. Stained cells were quantified manually or by ImageJ software (NIH). Probability (P) values were calculated using Student's t-test for comparison between two samples.

### Chromatin immunoprecipitation (ChIP)

ChIP experiments were performed according to EZ ChIP, Chromatin Immuno-precipitation Kit's Instruction Manual (Upstate, Millipore). In short, iGmnn ES cells were cultured in ES-CM on gelatin-coated plates and were treated with/without Tx. 10^6^ ES cells were used for one ChIP reaction. Cells were fixed in 1% formaldehyde in phosphate buffered saline for 10 min. Subsequently, cells were lysed in 1% SDS buffer (1% SDS, 10mM EDTA, 50 mM Tris, pH 8.1), and the chromatin shearing was performed using the Bioruptor XL sonicator (Diagenode) at 4°C to obtain 200–600 bp DNA fragments. Antibodies against Histone 3 (Abcam), Histone 3 lysine 4 trimethylation, Histone 3 lysine 27 trimethylation (Active motif), Histone 4 hyperacetylation (Millipore), Ezh2 (Cell signallig), Brg1 and Rabbit IgG (Santa Cruz Biotechnologies) were used for immunoprecipitation of pre-cleared chromatin. The complexes were eluted from washed protein A/G agarose beads (Santa Cruz). After reversal of crosslinking the DNA was purified using the QIAquick PCR purification kit (Qiagen), and qPCR reactions were performed. The primers used in the qPCR reactions are described in table S1.

## Supporting Information

Figure S1 **Targeting strategy to generate geminin conditional knockout allele.** A) Two LoxP sites were inserted in the first and third introns of geminin genomic locus upon site-specific recombination. The floxed allele possesses exon 2 and 3 flanked by LoxP sites and upon Cre mediated recombination exons 2 and 3 are excised. Thus the remaining conditional knockout allele loses its ability to produce functional protein (adapted after [55]). **B**) Southern blot analysis of t*he Gmnn^fl/+^* ESCs showing correct integration by indicated restriction enzymes. **C**) iGmnn ESCs were cultured for 72 hours in ES-CM and were treated for different periods of time with tamoxifen. The genomic DNA was extracted and genotyped. Different combinations of primers in separate reactions were used to amplify the floxed and recombined knockout alleles. The same amount of genomic DNA was used for each reaction. **D**) iGmnn ESCs were cultured for 96 hours in ES-CM and were treated for different periods of time with tamoxifen. The amount of geminin in whole cell lysates were anlaysed by western blot band quantified by imageJ.
(TIF)

Figure S2 **Cell cycle begins to lengthen after induction of geminin recombination.** A) iGmnn ESCs were treated with tamoxifen for 48 hours and stained for phosphor-histone 3 and TUNEL. The nuclei were stained with DAPI. **B**) iGmnn ESCs were treated with tamoxifen for 48 hours and prepared for flow cytometry of DNA content. The chart represents the cell cycle distribution of the cells.
(TIF)

Figure S3 **Geminin deficient ESCs don**'**t express trophoblastic, neuroectodermal and mesendodermal markers.** A) iGmnn ESCs were treated with tamoxifen for 48 hours immunostained for differentiation markers. The nuclei were stained with DAPI and the white bar represents 250 μm. **B**) iGmnn ESCs were differentiated for 4–6 days and were immunostained for differentiation markers. The white bar represents 100 μm. As shown the same concentration of primary and secondary antibodies detects positive cells for differentiation markers. **C**) wild type E3.5 blastocysts were grown on feeder layer in ES-CM in order to hatch and form outgrowths. The hatched blastocysts were positively stained for Trophoblastic markers Cdx2 and Troma-I in order to verify the reactivity of the antibodies and the sensitivity of our stainings.
(TIF)

Figure S4 **Geminin deficiency does not affect the Oct4 enhancer region.** ChIP-qPCR assays epigenetic marks binding at genomic locus of Oct4 gene. Oct4 genomic locus, analyzed fragments of the DNA have been marked with red, DE: Oct4 distal enhancer region, PE: Oct4 proximal enhancer region. Histone 3 ChIP, histone 4 hyper-acetylation (H4Ac) ChIP, histone 3 lysine 27 tri-methylation (H3K27me3) ChIP, Ezh2 ChIP and Brg1 ChIP in tamoxifen treated iGmnn cells and untreated iGmnn ESCs. Each sample is normalized to input, and error bars represent ± standard error of the mean (SEM) of biological triplicates. The X-axis represents positions relative to the transcriptional start site.
(TIF)

Figure S5 **Loss of Geminin does not cause cell cycle aberrations or apoptosis in MEFs.** A) Gmnn ^fl/fl^; ER-Cre and Gmnn ^fl/+^; ER-Cre MEFs were treated with tamoxifen for 48 hours. Whole cell lysate was run on the SDS-PAGE gels and geminin was immunobloted. The amount of loaded protein was controlled by Tubulin. **B**) fl/+ and fl/fl MEFs were treated with tamoxifen for 48 hours, and analyzed with flow cytometry. **C**) fl/+ and fl/fl MEFs were treated with tamoxifen for 48 hours and immuno-stained for phosho-histone 3, the M phase marker. In addition to tamoxifen MEFs received a 4 hours pulse of BrdU to label the cells in the S phase and were stained for BrdU in order to visualize the S phase. **D**) fl/+ and fl/fl MEFs were treated with tamoxifen for 48 hours and immuno-stained for cyclins. Cells were counted and abundances were calculated relative to total number of the cells. **E**) fl/+ and fl/fl MEFs were treated with tamoxifen for 48 hours and immuno-stained for Ki67,a marker for proliferating cells. Cells were counted and abundances were calculated relative to total number of the cells. **F**) fl/+ and fl/fl MEFs were treated with tamoxifen for 48 hours and stained for TUNEL (apoptosis marker). Treated cells were counted and the percentage of positive cells is represented in the graph.
(TIF)

Figure S6 **No efficient replacement of reprogramming factors by geminin.** Wild type MEFs were reprogrammed with viral particles containing different combinations of reprogramming factors (OSKM) and geminin (G). Transduced plates were stained for alkaline phosphatase 14 days after transduction.
(TIF)

Text S1 **Including the supplementary materials and methods, describing differentiation of ES cells, terminal deoxynucleotidyl transferase dUTP nick labeling (TUNEL) assay, BrdU staining, visualization of alkaline phosphatase activity, reprogramming, flow cytometric analysis, chimera analysis, quantitative RT-PCR, protein preparation and western blot analysis.**
(DOCX)

Table S1 **Listing the primers for quantitative RT-PCR and ChIP-qPCR.**
(DOCX)

## References

[1] Dejosez M, Zwaka TP (2012) Pluripotency and nuclear reprogramming. Annu Rev Biochem 81: 737–765. 22443931 10.1146/annurev-biochem-052709-104948

[2] Niwa H (2007) How is pluripotency determined and maintained? Development 134: 635–646. 17215298 10.1242/dev.02787

[3] Thomson M, Liu SJ, Zou LN, Smith Z, Meissner A, et al (2011) Pluripotency factors in embryonic stem cells regulate differentiation into germ layers. Cell 145: 875–889. 21663792 10.1016/j.cell.2011.05.017 PMC5603300

[4] Kroll KL, Salic AN, Evans LM, Kirschner MW (1998) Geminin, a neuralizing molecule that demarcates the future neural plate at the onset of gastrulation. Development 125: 3247–3258. 9671596 10.1242/dev.125.16.3247

[5] Seo S, Herr A, Lim JW, Richardson GA, Richardson H, et al (2005) Geminin regulates neuronal differentiation by antagonizing Brg1 activity. Genes Dev 19: 1723–1734. 16024661 10.1101/gad.1319105 PMC1176010

[6] Yellajoshyula D, Patterson ES, Elitt MS, Kroll KL (2011) Geminin promotes neural fate acquisition of embryonic stem cells by maintaining chromatin in an accessible and hyperacetylated state. Proc Natl Acad Sci U S A 108: 3294–3299. 21300881 10.1073/pnas.1012053108 PMC3044367

[7] Yellajoshyula D, Lim JW, Thompson DM Jr, Witt JS, Patterson ES, et al (2012) Geminin regulates the transcriptional and epigenetic status of neuronal fate-promoting genes during mammalian neurogenesis. Mol Cell Biol 32: 4549–4560. 22949506 10.1128/MCB.00737-12 PMC3486176

[8] Lim JW, Hummert P, Mills JC, Kroll KL (2011) Geminin cooperates with Polycomb to restrain multi-lineage commitment in the early embryo. Development 138: 33–44. 21098561 10.1242/dev.059824 PMC2998164

[9] Papanayotou C, Mey A, Birot AM, Saka Y, Boast S, et al (2008) A mechanism regulating the onset of Sox2 expression in the embryonic neural plate. PLoS Biol 6: e2. 10.1371/journal.pbio.0060002 PMC2174969 18184035

[10] Luo L, Yang X, Takihara Y, Knoetgen H, Kessel M (2004) The cell-cycle regulator geminin inhibits Hox function through direct and polycomb-mediated interactions. Nature 427: 749–753. 14973489 10.1038/nature02305

[11] McGarry TJ, Kirschner MW (1998) Geminin, an inhibitor of DNA replication, is degraded during mitosis. Cell 93: 1043–1053. 9635433 10.1016/s0092-8674(00)81209-x

[12] Petropoulou C, Kotantaki P, Karamitros D, Taraviras S (2008) Cdt1 and Geminin in cancer: markers or triggers of malignant transformation? Front Biosci 13: 4485–4494. 18508524 10.2741/3018

[13] Arias EE, Walter JC (2007) Strength in numbers: preventing rereplication via multiple mechanisms in eukaryotic cells. Genes Dev 21: 497–518. 17344412 10.1101/gad.1508907

[14] Hara K, Nakayama KI, Nakayama K (2006) Geminin is essential for the development of preimplantation mouse embryos. Genes to cells: devoted to molecular & cellular mechanisms 11: 1281–1293. 17054725 10.1111/j.1365-2443.2006.01019.x

[15] Gonzalez MA, Tachibana KE, Adams DJ, van der Weyden L, Hemberger M, et al (2006) Geminin is essential to prevent endoreduplication and to form pluripotent cells during mammalian development. Genes & development 20: 1880–1884. 16847348 10.1101/gad.379706 PMC1522086

[16] Karamitros D, Kotantaki P, Lygerou Z, Veiga-Fernandes H, Pachnis V, et al (2010) Differential geminin requirement for proliferation of thymocytes and mature T cells. J Immunol 184: 2432–2441. 20107189 10.4049/jimmunol.0901983

[17] Shinnick KM, Eklund EA, McGarry TJ (2010) Geminin deletion from hematopoietic cells causes anemia and thrombocytosis in mice. J Clin Invest 120: 4303–4315. 21041951 10.1172/JCI43556 PMC2993593

[18] Barry KA, Schultz KM, Payne CJ, McGarry TJ (2012) Geminin is required for mitotic proliferation of spermatogonia. Dev Biol 371: 35–46. 22898305 10.1016/j.ydbio.2012.07.031

[19] Spella M, Kyrousi C, Kritikou E, Stathopoulou A, Guillemot F, et al (2011) Geminin regulates cortical progenitor proliferation and differentiation. Stem Cells 29: 1269–1282. 21681860 10.1002/stem.678

[20] Schultz KM, Banisadr G, Lastra RO, McGuire T, Kessler JA, et al (2011) Geminin-deficient neural stem cells exhibit normal cell division and normal neurogenesis. PLoS One 6: e17736. 21408022 10.1371/journal.pone.0017736 PMC3052383

[21] Gonzalez MA, Tachibana KE, Adams DJ, van der Weyden L, Hemberger M, et al (2006) Geminin is essential to prevent endoreduplication and to form pluripotent cells during mammalian development. Genes Dev 20: 1880–1884. 16847348 10.1101/gad.379706 PMC1522086

[22] Yang VS, Carter SA, Hyland SJ, Tachibana-Konwalski K, Laskey RA, et al (2011) Geminin escapes degradation in G1 of mouse pluripotent cells and mediates the expression of Oct4, Sox2, and Nanog. Curr Biol 21: 692–699. 21497086 10.1016/j.cub.2011.03.026 PMC3083515

[23] Fujii-Yamamoto H, Kim JM, Arai K, Masai H (2005) Cell cycle and developmental regulations of replication factors in mouse embryonic stem cells. J Biol Chem 280: 12976–12987. 15659392 10.1074/jbc.M412224200

[24] Hayashi S, McMahon AP (2002) Efficient recombination in diverse tissues by a tamoxifen-inducible form of Cre: a tool for temporally regulated gene activation/inactivation in the mouse. Dev Biol 244: 305–318. 11944939 10.1006/dbio.2002.0597

[25] Gaspard N, Bouschet T, Herpoel A, Naeije G, van den Ameele J, et al (2009) Generation of cortical neurons from mouse embryonic stem cells. Nat Protoc 4: 1454–1463. 19798080 10.1038/nprot.2009.157

[26] Tomioka M, Nishimoto M, Miyagi S, Katayanagi T, Fukui N, et al (2002) Identification of Sox-2 regulatory region which is under the control of Oct-3/4-Sox-2 complex. Nucleic Acids Res 30: 3202–3213. 12136102 10.1093/nar/gkf435 PMC135755

[27] Yeom YI, Fuhrmann G, Ovitt CE, Brehm A, Ohbo K, et al (1996) Germline regulatory element of Oct-4 specific for the totipotent cycle of embryonal cells. Development 122: 881–894. 8631266 10.1242/dev.122.3.881

[28] Ho L, Jothi R, Ronan JL, Cui K, Zhao K, et al (2009) An embryonic stem cell chromatin remodeling complex, esBAF, is an essential component of the core pluripotency transcriptional network. Proc Natl Acad Sci U S A 106: 5187–5191. 19279218 10.1073/pnas.0812888106 PMC2654397

[29] Takahashi K, Yamanaka S (2006) Induction of pluripotent stem cells from mouse embryonic and adult fibroblast cultures by defined factors. Cell 126: 663–676. 16904174 10.1016/j.cell.2006.07.024

[30] Stadtfeld M, Maherali N, Breault DT, Hochedlinger K (2008) Defining molecular cornerstones during fibroblast to iPS cell reprogramming in mouse. Cell Stem Cell 2: 230–240. 18371448 10.1016/j.stem.2008.02.001 PMC3538379

[31] Ichida JK, Blanchard J, Lam K, Son EY, Chung JE, et al (2009) A small-molecule inhibitor of tgf-Beta signaling replaces sox2 in reprogramming by inducing nanog. Cell Stem Cell 5: 491–503. 19818703 10.1016/j.stem.2009.09.012 PMC3335195

[32] Zhu W, Depamphilis ML (2009) Selective killing of cancer cells by suppression of geminin activity. Cancer Res 69: 4870–4877. 19487297 10.1158/0008-5472.CAN-08-4559 PMC2749580

[33] Ballabeni A, Park IH, Zhao R, Wang W, Lerou PH, et al (2011) Cell cycle adaptations of embryonic stem cells. Proc Natl Acad Sci U S A 108: 19252–19257. 22084091 10.1073/pnas.1116794108 PMC3228440

[34] Kalaszczynska I, Geng Y, Iino T, Mizuno S, Choi Y, et al (2009) Cyclin A is redundant in fibroblasts but essential in hematopoietic and embryonic stem cells. Cell 138: 352–365. 19592082 10.1016/j.cell.2009.04.062 PMC2745999

[35] Menchon C, Edel MJ, Izpisua Belmonte JC (2011) The cell cycle inhibitor p27Kip(1) controls self-renewal and pluripotency of human embryonic stem cells by regulating the cell cycle, Brachyury and Twist. Cell Cycle 10: 1435–1447. 21478681 10.4161/cc.10.9.15421 PMC3685623

[36] Ruiz S, Panopoulos AD, Herrerias A, Bissig KD, Lutz M, et al (2011) A high proliferation rate is required for cell reprogramming and maintenance of human embryonic stem cell identity. Curr Biol 21: 45–52. 21167714 10.1016/j.cub.2010.11.049 PMC3034649

[37] Li VC, Ballabeni A, Kirschner MW (2012) Gap 1 phase length and mouse embryonic stem cell self-renewal. Proc Natl Acad Sci U S A 109: 12550–12555. 22802651 10.1073/pnas.1206740109 PMC3412034

[38] Filipczyk AA, Laslett AL, Mummery C, Pera MF (2007) Differentiation is coupled to changes in the cell cycle regulatory apparatus of human embryonic stem cells. Stem Cell Res 1: 45–60. 19383386 10.1016/j.scr.2007.09.002

[39] White J, Stead E, Faast R, Conn S, Cartwright P, et al (2005) Developmental activation of the Rb-E2F pathway and establishment of cell cycle-regulated cyclin-dependent kinase activity during embryonic stem cell differentiation. Mol Biol Cell 16: 2018–2027. 15703208 10.1091/mbc.E04-12-1056 PMC1073679

[40] Lessard J, Wu JI, Ranish JA, Wan M, Winslow MM, et al (2007) An essential switch in subunit composition of a chromatin remodeling complex during neural development. Neuron 55: 201–215. 17640523 10.1016/j.neuron.2007.06.019 PMC2674110

[41] Pasini D, Cloos PA, Walfridsson J, Olsson L, Bukowski JP, et al (2010) JARID2 regulates binding of the Polycomb repressive complex 2 to target genes in ES cells. Nature 464: 306–310. 20075857 10.1038/nature08788

[42] Gao Z, Zhang J, Bonasio R, Strino F, Sawai A, et al (2012) PCGF homologs, CBX proteins, and RYBP define functionally distinct PRC1 family complexes. Mol Cell 45: 344–356. 22325352 10.1016/j.molcel.2012.01.002 PMC3293217

[43] Ho L, Ronan JL, Wu J, Staahl BT, Chen L, et al (2009) An embryonic stem cell chromatin remodeling complex, esBAF, is essential for embryonic stem cell self-renewal and pluripotency. Proc Natl Acad Sci U S A 106: 5181–5186. 19279220 10.1073/pnas.0812889106 PMC2654396

[44] Luo L, Uerlings Y, Happel N, Asli NS, Knoetgen H, et al (2007) Regulation of geminin functions by cell cycle-dependent nuclear-cytoplasmic shuttling. Mol Cell Biol 27: 4737–4744. 17470552 10.1128/MCB.00123-07 PMC1951490

[45] Boos A, Lee A, Thompson DM, Kroll KL (2006) Subcellular translocation signals regulate Geminin activity during embryonic development. Biol Cell 98: 363–375. 16464175 10.1042/BC20060007

[46] Kulartz M, Knippers R (2004) The replicative regulator protein geminin on chromatin in the HeLa cell cycle. J Biol Chem 279: 41686–41694. 15284237 10.1074/jbc.M405798200

[47] Pitulescu ME, Teichmann M, Luo L, Kessel M (2009) TIPT2 and geminin interact with basal transcription factors to synergize in transcriptional regulation. BMC Biochem 10: 16. 19515240 10.1186/1471-2091-10-16 PMC2702275

[48] Ho L, Miller EL, Ronan JL, Ho WQ, Jothi R, et al (2011) esBAF facilitates pluripotency by conditioning the genome for LIF/STAT3 signalling and by regulating polycomb function. Nat Cell Biol 13: 903–913. 21785422 10.1038/ncb2285 PMC3155811

[49] Avilion AA, Nicolis SK, Pevny LH, Perez L, Vivian N, et al (2003) Multipotent cell lineages in early mouse development depend on SOX2 function. Genes Dev 17: 126–140. 12514105 10.1101/gad.224503 PMC195970

[50] Masui S, Nakatake Y, Toyooka Y, Shimosato D, Yagi R, et al (2007) Pluripotency governed by Sox2 via regulation of Oct3/4 expression in mouse embryonic stem cells. Nat Cell Biol 9: 625–635. 17515932 10.1038/ncb1589

[51] Bergsland M, Ramskold D, Zaouter C, Klum S, Sandberg R, et al (2011) Sequentially acting Sox transcription factors in neural lineage development. Genes Dev 25: 2453–2464. 22085726 10.1101/gad.176008.111 PMC3243056

[52] Bylund M, Andersson E, Novitch BG, Muhr J (2003) Vertebrate neurogenesis is counteracted by Sox1-3 activity. Nat Neurosci 6: 1162–1168. 14517545 10.1038/nn1131

[53] Wang Z, Oron E, Nelson B, Razis S, Ivanova N (2012) Distinct lineage specification roles for NANOG, OCT4, and SOX2 in human embryonic stem cells. Cell Stem Cell 10: 440–454. 22482508 10.1016/j.stem.2012.02.016

[54] Buganim Y, Faddah DA, Cheng AW, Itskovich E, Markoulaki S, et al (2012) Single-cell expression analyses during cellular reprogramming reveal an early stochastic and a late hierarchic phase. Cell 150: 1209–1222. 22980981 10.1016/j.cell.2012.08.023 PMC3457656

[55] Kopp JL, Ormsbee BD, Desler M, Rizzino A (2008) Small increases in the level of Sox2 trigger the differentiation of mouse embryonic stem cells. Stem Cells 26: 903–911. 18238855 10.1634/stemcells.2007-0951

[56] Uerlings Y (2008) Die Funktion von Geminin beim Übergang von Neuro- zu Gliogenese in der Maus. der Georg-August-Universität zu Göttingen: der Georg-August-Universität zu Göttingen.

[57] Liu P, Jenkins NA, Copeland NG (2003) A highly efficient recombineering-based method for generating conditional knockout mutations. Genome Res 13: 476–484. 12618378 10.1101/gr.749203 PMC430283

[58] Voss AK, Thomas T, Gruss P (1997) Germ line chimeras from female ES cells. Exp Cell Res 230: 45–49. 9013705 10.1006/excr.1996.3418

[59] Wiese C, Kania G, Rolletschek A, Blyszczuk P, Wobus AM (2006) Pluripotency: capacity for in vitro differentiation of undifferentiated embryonic stem cells. Methods Mol Biol 325: 181–205. 16761727 10.1385/1-59745-005-7:181

