# Supplementary figures and images for: Geminin Is Required for the Maintenance of Pluripotency
Source: PLoS One. 2013 Sep 19;8(9):e73826. doi: 10.1371/journal.pone.0073826 (PMC3777968; doi:10.1371/journal.pone.0073826)

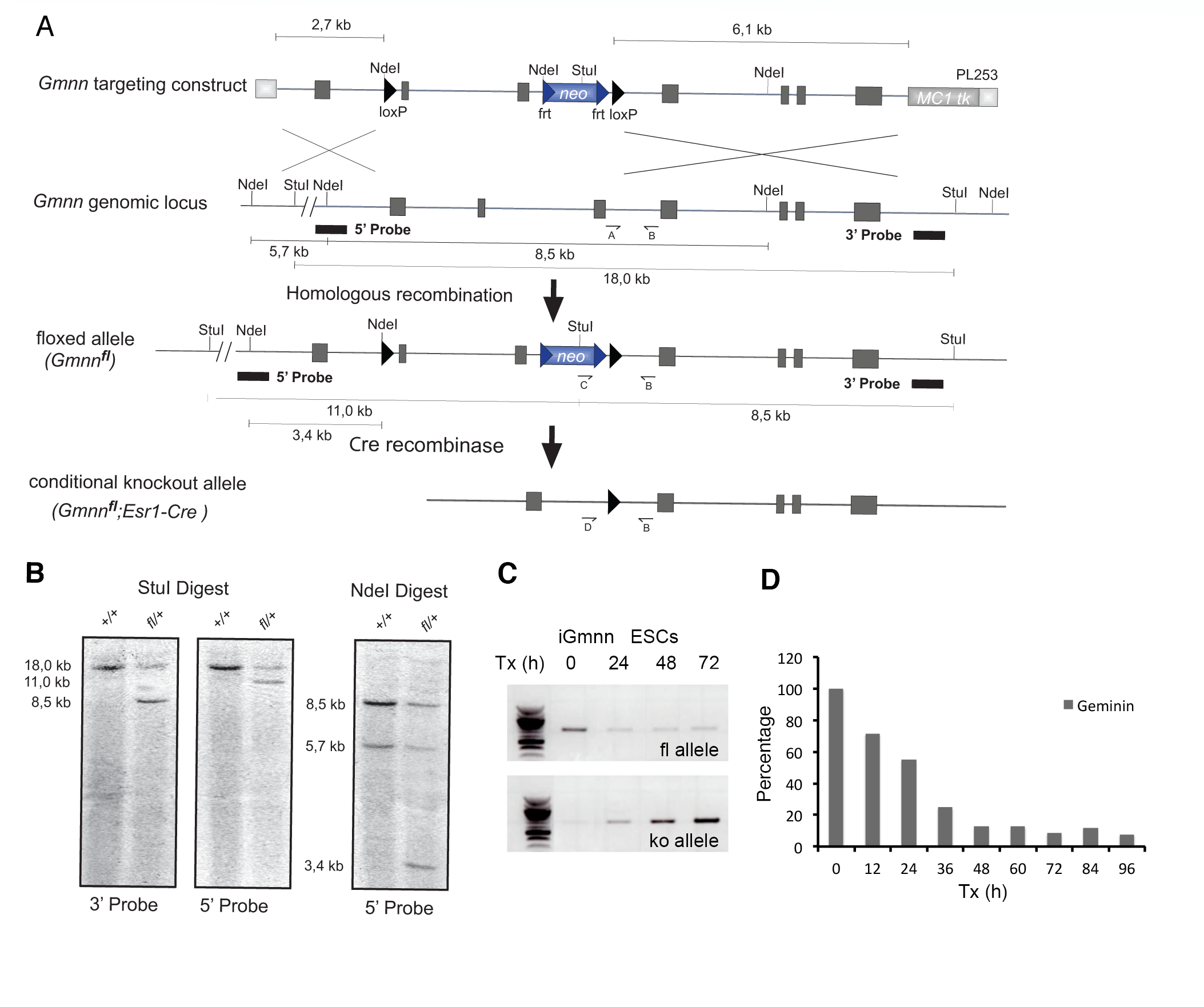

Supplement: Figure S1 — Targeting strategy to generate geminin conditional knockout allele. A) Two LoxP sites were inserted in the first and third introns of geminin genomic locus upon site-specific recombination. The floxed allele possesses exon 2 and 3 flanked by LoxP sites and upon Cre mediated recombination exons 2 and 3 are excised. Thus the remaining conditional knockout allele loses its ability to produce functional protein (adapted after [55]). B) Southern blot analysis of the Gmnnfl/+ ESCs showing correct integration by indicated restriction enzymes. C) iGmnn ESCs were cultured for 72 hours in ES-CM and were treated for different periods of time with tamoxifen. The genomic DNA was extracted and genotyped. Different combinations of primers in separate reactions were used to amplify the floxed and recombined knockout alleles. The same amount of genomic DNA was used for each reaction. D) iGmnn ESCs were cultured for 96 hours in ES-CM and were treated for different periods of time with tamoxifen. The amount of geminin in whole cell lysates were anlaysed by western blot band quantified by imageJ. (TIF) [file pone.0073826.s001.tif]

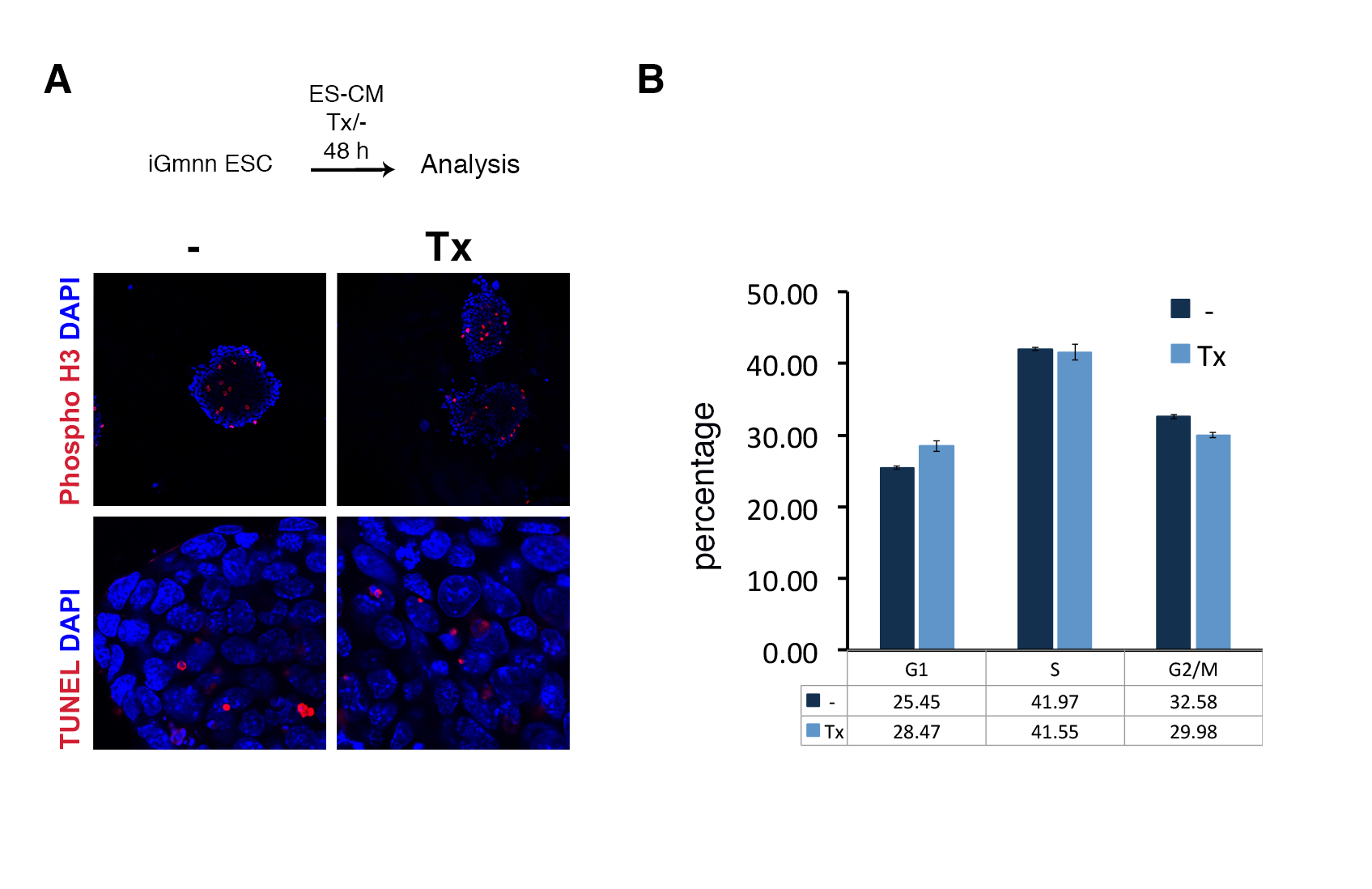

Supplement: Figure S2 — Cell cycle begins to lengthen after induction of geminin recombination. A) iGmnn ESCs were treated with tamoxifen for 48 hours and stained for phosphor-histone 3 and TUNEL. The nuclei were stained with DAPI. B) iGmnn ESCs were treated with tamoxifen for 48 hours and prepared for flow cytometry of DNA content. The chart represents the cell cycle distribution of the cells. (TIF) [file pone.0073826.s002.tif]

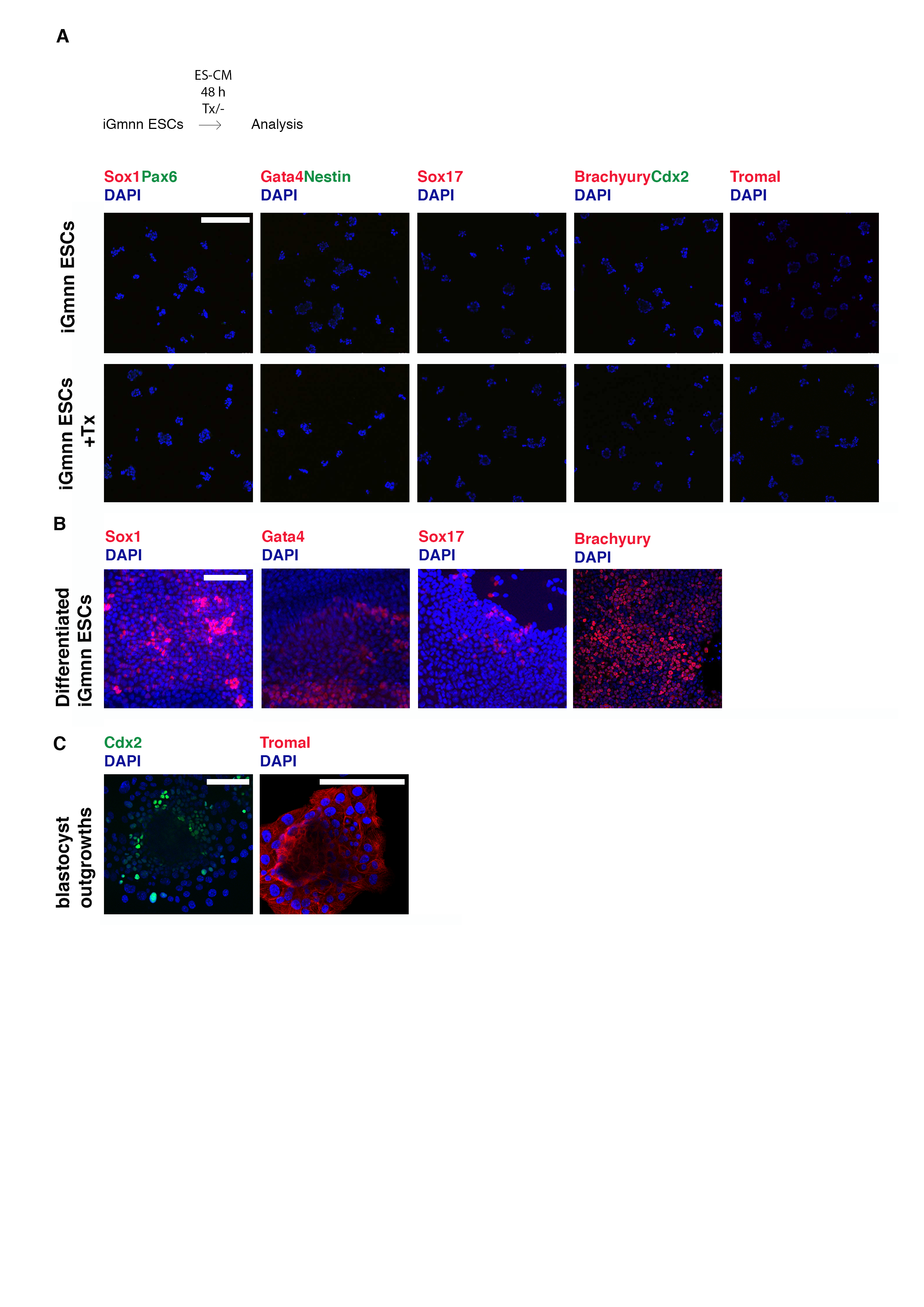

Supplement: Figure S3 — Geminin deficient ESCs don't express trophoblastic, neuroectodermal and mesendodermal markers. A) iGmnn ESCs were treated with tamoxifen for 48 hours immunostained for differentiation markers. The nuclei were stained with DAPI and the white bar represents 250 μm. B) iGmnn ESCs were differentiated for 4–6 days and were immunostained for differentiation markers. The white bar represents 100 μm. As shown the same concentration of primary and secondary antibodies detects positive cells for differentiation markers. C) wild type E3.5 blastocysts were grown on feeder layer in ES-CM in order to hatch and form outgrowths. The hatched blastocysts were positively stained for Trophoblastic markers Cdx2 and Troma-I in order to verify the reactivity of the antibodies and the sensitivity of our stainings. (TIF) [file pone.0073826.s003.tif]

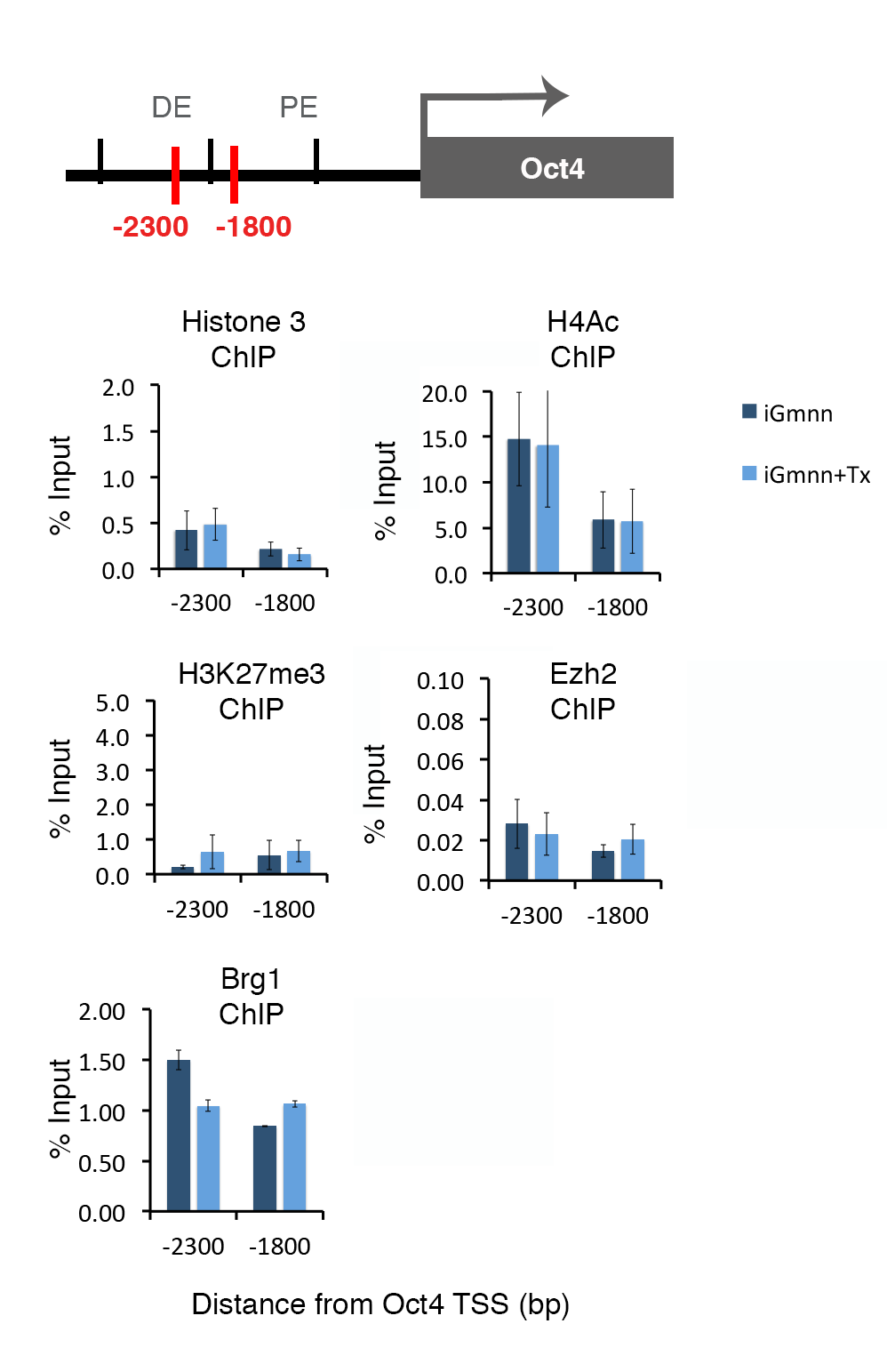

Supplement: Figure S4 — Geminin deficiency does not affect the Oct4 enhancer region. ChIP-qPCR assays epigenetic marks binding at genomic locus of Oct4 gene. Oct4 genomic locus, analyzed fragments of the DNA have been marked with red, DE: Oct4 distal enhancer region, PE: Oct4 proximal enhancer region. Histone 3 ChIP, histone 4 hyper-acetylation (H4Ac) ChIP, histone 3 lysine 27 tri-methylation (H3K27me3) ChIP, Ezh2 ChIP and Brg1 ChIP in tamoxifen treated iGmnn cells and untreated iGmnn ESCs. Each sample is normalized to input, and error bars represent ± standard error of the mean (SEM) of biological triplicates. The X-axis represents positions relative to the transcriptional start site. (TIF) [file pone.0073826.s004.tif]

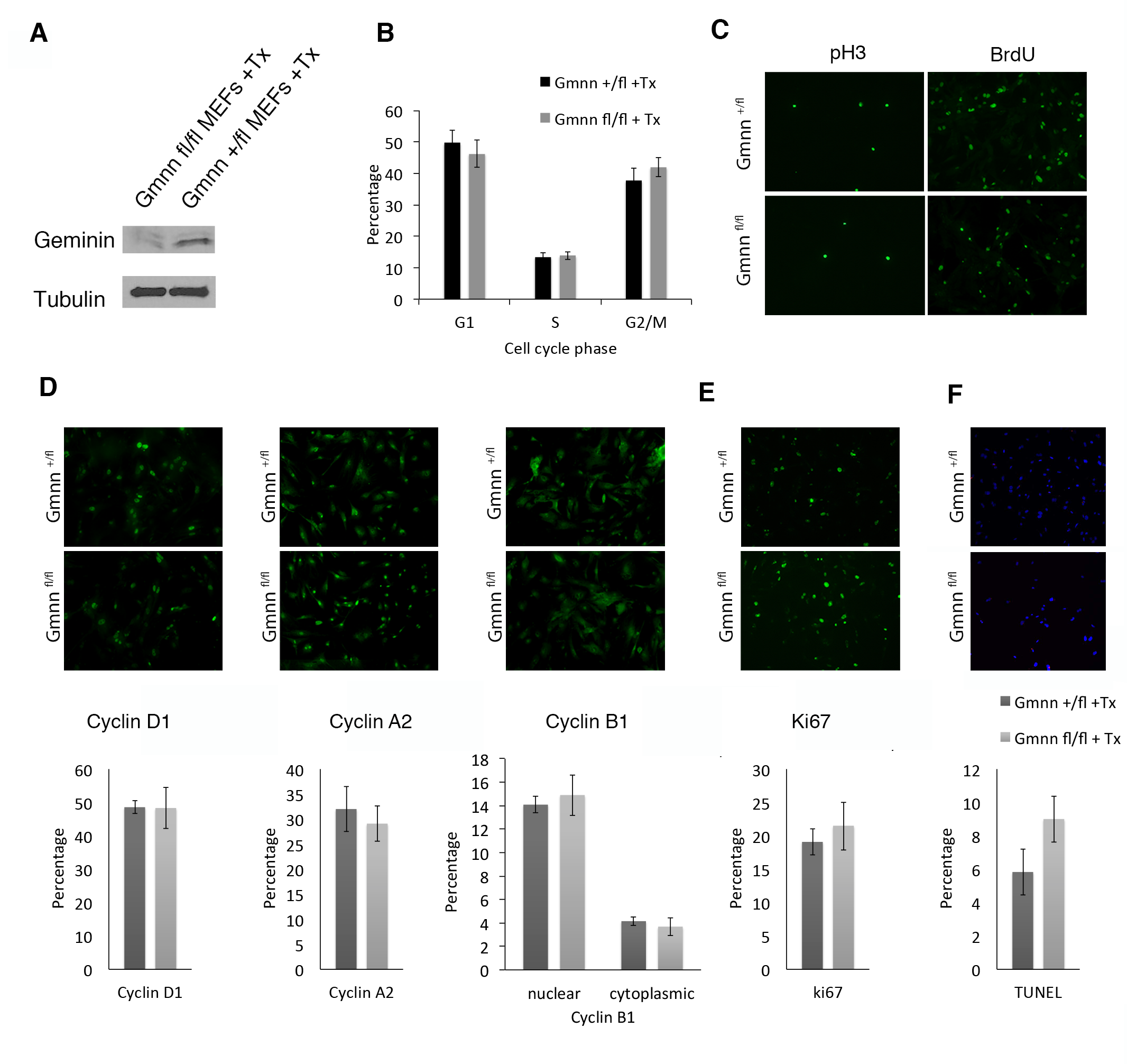

Supplement: Figure S5 — Loss of Geminin does not cause cell cycle aberrations or apoptosis in MEFs. A) Gmnn fl/fl; ER-Cre and Gmnn fl/+; ER-Cre MEFs were treated with tamoxifen for 48 hours. Whole cell lysate was run on the SDS-PAGE gels and geminin was immunobloted. The amount of loaded protein was controlled by Tubulin. B) fl/+ and fl/fl MEFs were treated with tamoxifen for 48 hours, and analyzed with flow cytometry. C) fl/+ and fl/fl MEFs were treated with tamoxifen for 48 hours and immuno-stained for phosho-histone 3, the M phase marker. In addition to tamoxifen MEFs received a 4 hours pulse of BrdU to label the cells in the S phase and were stained for BrdU in order to visualize the S phase. D) fl/+ and fl/fl MEFs were treated with tamoxifen for 48 hours and immuno-stained for cyclins. Cells were counted and abundances were calculated relative to total number of the cells. E) fl/+ and fl/fl MEFs were treated with tamoxifen for 48 hours and immuno-stained for Ki67,a marker for proliferating cells. Cells were counted and abundances were calculated relative to total number of the cells. F) fl/+ and fl/fl MEFs were treated with tamoxifen for 48 hours and stained for TUNEL (apoptosis marker). Treated cells were counted and the percentage of positive cells is represented in the graph. (TIF) [file pone.0073826.s005.tif]

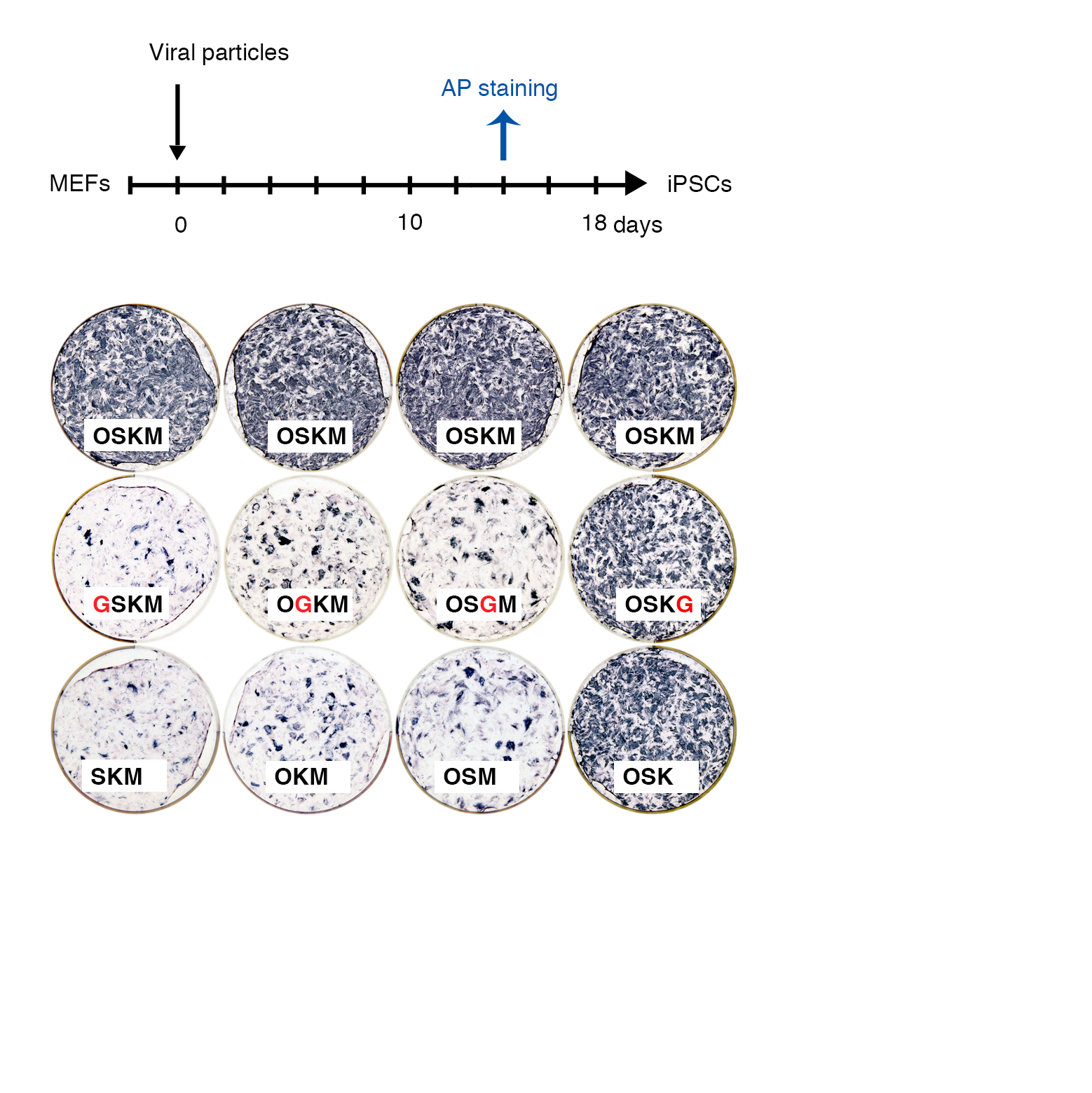

Supplement: Figure S6 — No efficient replacement of reprogramming factors by geminin. Wild type MEFs were reprogrammed with viral particles containing different combinations of reprogramming factors (OSKM) and geminin (G). Transduced plates were stained for alkaline phosphatase 14 days after transduction. (TIF) [file pone.0073826.s006.tif]
