# Supplementary material for: Geminin Is Required for the Maintenance of Pluripotency
Source: PLoS One. 2013 Sep 19;8(9):e73826. doi: 10.1371/journal.pone.0073826 (PMC3777968; doi:10.1371/journal.pone.0073826)
Supplement: Text S1 — Including the supplementary materials and methods, describing differentiation of ES cells, terminal deoxynucleotidyl transferase dUTP nick labeling (TUNEL) assay, BrdU staining, visualization of alkaline phosphatase activity, reprogramming, flow cytometric analysis, chimera analysis, quantitative RT-PCR, protein preparation and western blot analysis. (DOCX) [file pone.0073826.s007.docx]

**Supplementary Methods:**

**Differentiation of ES cells**

ESCs were differentiated using three different methods; I- Embryoid Body differentiation: ESCs were plated in non-adhesive culture plates in density of 2 x 10^5^ cells/ml differentiation medium (DM): IMDM (Gibco) supplemented with 20% FBS (PANbiotech), 450 nM monothioglycerol (Sigma-Aldrich), 2 mM L-Glutamine (Gibco) and 1% non essential amino acids (Gibco). The EBs were formed and cultured further for 5 days. Then, EBs were plated on adhesive culture plates for 4 more days [58]. II- Monolayer differentiation: ESCs were trypsinized into single cells and 1.5 x 10^4^ cells/cm^2^ were transferred into gelatin coated culture plates or glass chamber slides. These plates were cultured further for 4 days in differentiation medium. III- Differentiation toward neuroectoderm and mesendoderm progenitors: ESCs were differentiated as described previously [3,25] in default defined medium (DDM).

**Terminal deoxynucleotidyl transferase dUTP nick labeling (TUNEL) assay**

Apoptotic cells were detected using the ApopTag® Plus Fluorescein In Situ Apoptosis Detection Kit (Chemicon, Millipore). Percentages were determined by counting the number of the TUNEL positive cells in comparison to DAPI stained nuclei.

**BrdU staining**

In order to visualize the replicating cells in the S-phase of the cell cycle, the cells were incubated with bromodeoxyuridine (BrdU) 4 hours to overnight, then were fixed and immunostained with anti-BrdU antibody (1:25, Roche). Percentage of S-phase was determined by counting the number of the BrdU positive cells in comparison to DAPI stained nuclei.

**Visualization of alkaline phosphatase activity**

The culture plates containing ESCs or reprogrammed MEFs were washed once with PBS and were fixed in 4% paraformaldehyde in PBS for 30 min. The plates were washed three times with PBS and once with alkaline phosphatase buffer (100 mM Tris-HCl pH 9.5, 100 mM NaCl and 50 mM MgCl2). The cells were stained in blue by exposure to NBT/BCIP working solution (2% NBT/BCIP enzyme substrate (Roche) in alkaline phosphatase buffer) for 10-30 minutes in dark.

**Reprogramming**

PlatE cells (Platinum-E retroviral packaging cell line, Ecotrophic, Cell Biolabs, Inc.) were maintained in 1 μg/ml Puromycin and 10 μg/ml Blasticidin. For production of viral particles the cells were transfected with Fugene 6™ (Promega) and one of the pmXs plasmids: pMXs-Oct3/4 (Addgene-plasmid 13366), pMXs-Sox2 (Addgene-plasmid 13367), pMXs-Klf4 (Addgene-plasmid 13370), pMXs-c-Myc (Addgene-plasmid 13375), pMXs-Gmnn. Transfection was done with Fugene 6™ (Promega) according to instruction provided by manufacturer. 36 hours later the viral particles have been collected and used for transfection of primary mouse fibroblasts. In order to obtain OSKM (Oct4, Sox2, Klf4 and c-Myc) viral particle containing medium, equal amounts of each supernatant was mixed together. Three-factor reprogramming (OSK, OSM, SKM and OKM) was done with combining one part fibroblast medium to three parts viral particle containing mediums. 2-3 days after transduction the cells were plated on feeder-coated plates in ES-CM. Medium was changed every 1-2 days depending on the cell proliferation rate.

**Flow cytometric analysis**

Cells were trypsinized into single and fixed with cold 75% ethanol. Shortly before the analysis, the cells were spun down, and re-suspended in PBS containing 100 μg/ml RNase A (Roche) and 50 μg/ml propidium iodide (Sigma-Aldrich). The staining was performed for 30-60 minutes at RT in the dark. The cells were analyzed for cell cycle distribution using FACSCalibur™ Flow Cytometer (BD biosciences). The collected data were analyzed using the FlowJo software (Tree Star Inc.).

**Chimera analysis**

Five to 8 dissociated ES cells were injected into C57Bl6/N & FVB/N 8-cell-stage embryos. Injected embryos were transferred to the uteri of CD1 females, previously mated with vasectomized males, at 2.5 dpc. Chimaeras were identified by coat color mosaicism or genotyping PCR of blood. Germline transmissibility was confirmed by mating of the chimeras with an appropriate wild type mouse.

**Quantitative RT-PCR**

Total RNA from cell cultures was isolated with RNeasy™ mini kit (Qiagen), and extracted RNA was treated with RNase free DNase (Qiagen). Reverse transcription was conducted with the Omniscript™ kit (Qiagen), according to the instruction provided by manufacturers. Real time quantitative PCR reactions were hold in triplicates with KAPA SYBR™ FAST qPCR Master Mix (PeqLab). Relative quantification was determined using the Pfaffl method. Primers are shown in supplementary material Table S1.

**Protein preparation and western blot analysis**

Whole cell extract was prepared using a modified RIPA buffer (50 mM Tris, pH 7.4, 1% NP-40, 0,25% Na-deoxycholate, 0.5 mM EDTA, 150 mM NaCl, 1 mM PMSF). Protein samples were mixed with loading buffer and electrophoresed on 12 or 15% acrylamide gels. The protein was transferred on nitrocellulose membrane. The membrane was blocked with blocking buffer (5% milk power in PBS-T), stained with appropriate primary antibody. The membrane was washed with PBS-T three times and incubated with the HRP-conjugated secondary antibody diluted in the blocking buffer for 2 h at room temprature. It was washed with PBS-T and the signal was detected using, SuperSignal® West pico Maximum Sensitivity Substrate (ThermoScientific) or homemade-ECL (100mM glycine pH 10 (with NaOH), 0.4mM luminol (Sigma-Aldrich), 8mM 4-iodophenol (Sigma-Aldrich), 0.12% (w/w) hydrogen peroxide). The western blot bands were quantified using ImageJ software (NIH).

**Antibodies for western blot analysis**

Primary antibodies were Geminin-FL209 (1:500), Sox2 (1:1000, Santa Cruz Biotechnologies), Nanog (1:1000, Cosmobio), Oct3/4 (1:1000, BD Bioscience), α-Tubulin (1:2000, Sigma-Aldrich). Appropriate secondary antibodies Conjugated with HRP (Invitrogen) were diluted 1:10000.
