# Supplementary material for: Geminin Is Required for the Maintenance of Pluripotency
Source: PLoS One. 2013 Sep 19;8(9):e73826. doi: 10.1371/journal.pone.0073826 (PMC3777968; doi:10.1371/journal.pone.0073826)
Supplement: Table S1 — Listing the primers for quantitative RT-PCR and ChIP-qPCR. (DOCX) [file pone.0073826.s008.docx]

**Table S1**. Primers used in this study.

| **Gene** |  | **Primer squence** |
| --- | --- | --- |
| Quantitative PCR | | |
| Geminin1 | forward | GCA GAG AAA ATG AGT TGC CAA |
|  | reverse | ACT CAG CCT CTC GAT TAC C |
| Geminin2 | forward | ACGCTGAAGATGATCCAGCCTTCT |
|  | reverse | TAGCTGGTCATCCCAAAGCTTCCT |
| Oct4 | forward | CTG AGG GCC AGG CAG GAG CAC GAG |
|  | reverse | CTG TAG GGA GGG CTT CGG GAC TT |
| Nanog | forward | CAC CCA CCC ATG CTA GTC TT |
|  | reverse | ACC CTC AAA CTC CTG GTC CT |
| Zfp42 | forward | TGT CCT CAG GCT GGG TAG TC |
|  | reverse | TGA TTT TCT GCC GTA TGC AA |
| Sox2 | forward | GGT TAC CTC TTC CTC CCA CTC CAG |
|  | reverse | TCA CAT GTG CGA CAG GGG CAG |
| Sox2-endo | forward | TAG AGC TAG ACT CCG GGC GA TGA |
|  | reverse | TTG CCT TAA ACA AGA CCA CGA AA |
| UbC | forward | AGGTCAAACAGGAAGACAGACGTA |
|  | reverse | TCACACCCAAGAACAAGCACA |
| Hprt | forward | GTC CTG TGG CCA TCT GCC TA |
|  | reverse | GGG ACG CAG CAA CTG ACA TT |
| Gapdh | forward | CCA TGT TTG TGA TGG GTG TGAACC |
|  | reverse | TGT GAG GGA GAT GCT CAG TGTTGG |
| Chromatin immunoprecipitation | | |
| -6900 Sox2 | Forward | CATAGCGTGTCAGTGATCTCC |
|  | Reverse | GCTTCCAAACCCATCCTTACAG |
| -4100 Sox2 | Forward | CCCTCCTCTCCTAATCTCCTTATGG |
|  | Reverse | AACTCTCATAGCCCTAACTGTC |
| -1600 Sox2 | Forward | GAGTTCCAGCTTTGCCTTTG |
|  | Reverse | TTGTTCCCAGCCTTTTCCTAG |
| 3500 Sox2 | Forward | CTCAGCCTCTAGGCCTGTGT |
|  | Reverse | CCCTTCCCAGTACCTTACCC |
| 3750 Sox2 | Forward | GCACAGTCGACAGTTCTTGC |
|  | Reverse | AGGCTGAGTCGGGTCAATTA |
| 4250 Sox2 | Forward | GATAAACTGCAGCGCTACCC |
|  | Reverse | CCTCGGAAAGAAGTCACAGG |
| 5500 Sox2 | Forward | CAAGGACAACTGCTAAACTGC |
|  | Reverse | GACAGACCGATAAGAGATGCC |
| -2300 Oct4 | Forward | AGGGCACATCTGTTTCAAGC |
|  | Reverse | CTGGCCAGGACAAGAGACAT |
| -1800 Oct4 | Forward | CTCTCGTCCTAGCCCTTCCT |
|  | Reverse | ATCTCTCTGGCCCTCTCCAT |
